# Integrated phenotyping reveals coordinated physiological responses to cold, freezing, and recovery in *Ramonda* resurrection plants

**DOI:** 10.1093/aobpla/plag046

**Published:** 2026-09-29

**Authors:** Fitim Kastrati, Bekim Gashi, Boris Lazarević

**Affiliations:** Laboratory of Plant Physiology, Department of Biology, Faculty of Mathematical and Natural Sciences, University of Prishtina, Eqerem Cabej str, No 51, Prishtina 10000, Kosovo; Laboratory of Plant Physiology, Department of Biology, Faculty of Mathematical and Natural Sciences, University of Prishtina, Eqerem Cabej str, No 51, Prishtina 10000, Kosovo; Department of Plant Nutrition, University of Zagreb Faculty of Agriculture, Svetošimunska cesta 25, 10000 Zagreb, Croatia; Centre of Excellence for Biodiversity and Molecular Plant Breeding (CroP-BioDiv), Svetošimunska cesta 25, 10000 Zagreb, Croatia

**Keywords:** resurrection plants, phenotyping methods, freezing-induced desiccation, recovery

## Abstract

Resurrection plants provide insights into the physiological responses that enable survival under extreme dehydration and low-temperature stress. This study presents an integrated phenotyping analysis of cold and freezing responses and subsequent recovery in *Ramonda serbica* and *Ramonda nathaliae*. Using chlorophyll fluorescence, multispectral reflectance and gas-exchange analysis, combined with regression modelling, we characterized changes in photosynthetic and water-related traits during sequential cooling, freezing-induced dehydration and rehydration. Both species underwent pronounced dehydration during freezing and showed recovery of water status and photosynthetic function following rehydration. Cooling and freezing were accompanied by declines in PSII efficiency and nonphotochemical quenching (NPQ), while gas-exchange measurements during cooling showed reductions in CO_2_ assimilation and stomatal conductance. During recovery, PSII efficiency increased towards pre-stress levels, whereas NPQ was rapidly reactivated, consistent with the restoration of regulated photoprotective energy dissipation. The two species showed similar responses in relative water content, PSII efficiency and multispectral traits, while species-dependent differences were most evident in NPQ and in the temporal dynamics of several gas-exchange traits. NDVI recovered to near pre-stress values, whereas specific green reflectance remained below initial levels at the end of the recovery period, indicating asynchronous recovery among spectral traits. Overall, the results indicate that *R. serbica* and *R. nathaliae* share reversible physiological responses to freezing-induced dehydration, with differences between species primarily in the timing and magnitude of particular recovery processes rather than fundamentally different tolerance strategies. Integrating phenotyping with temporal modelling provides a framework for identifying coordinated and asynchronous components of stress and recovery responses in resurrection plants.

## Teaser

Resurrection plants can survive extreme water loss, but less is known about how they cope with freezing and recover afterwards. We show that two Ramonda species undergo pronounced dehydration and reduced photosynthetic activity during freezing, yet largely restore water status and photosynthetic function after rehydration. Their overall responses were remarkably similar, with species differences mainly reflecting the timing and magnitude of specific recovery processes.

## Introduction

Resurrection plants are rare among vascular plants and are characterized by their ability to survive extreme desiccation. Among them, the Balkan endemics *Ramonda serbica* and *Ramonda nathaliae* are especially notable. They inhabit shaded, rocky substrates and have evolved strategies enabling tolerance to severe water deficit (Gashi et al. 2013, Legardón and García-Plazaola 2023). Their vegetative desiccation tolerance allows survival after losing more than 90% of cellular water (Gaff and Oliver 2013). During drying, metabolic activity is largely suspended, and LEA proteins, antioxidants and sugars stabilize cellular structures, whereas rehydration triggers rapid metabolic reactivation, including gene expression, membrane repair and protein synthesis (Georgieva et al. 2020, Oliver et al. 2020, Farrant and Hilhorst 2022).

Although desiccation tolerance in *Ramonda* sp. has been investigated, much less is known about their responses to low temperatures, particularly recovery after freezing-induced dehydration (Gashi et al. 2025). Given their exposure to fluctuating microclimates and occasional subzero temperatures, understanding freezing tolerance is essential and may offer insights relevant to improving stress resilience in crops (Rascio and Rocca 2005). Research remains limited due to the slow growth of these species and challenges in controlled experimentation (Legardón and García-Plazaola 2023).

Nonfreezing cold stress primarily affects chloroplast function, disrupting photosynthesis, enhancing ROS production and triggering photoinhibition (Fernández and Strand 2008). Freezing further restricts cellular water availability and induces ultrastructural changes (Mihailova et al. 2020, 2022). Ex situ studies on *R. serbica* and *R. nathaliae* show declines in PSII efficiency and stomatal conductance after freezing, alongside putatively adaptive responses such as enhanced thermal dissipation, downregulated photosynthesis and accumulation of dehydrins and ELIPs (Mihailova et al. 2023, Kastrati et al. 2025). Similar protective mechanisms have been reported in *Haberlea rhodopensis* (Georgieva et al. 2020).

Advanced noninvasive phenotyping has become valuable for studying such dynamic responses. Phenotyping methods, including chlorophyll fluorescence imaging, multispectral imaging combined with gas-exchange analysis, enable early detection of physiological disturbances and high-resolution monitoring of photosynthetic performance, stomatal behaviour and pigment dynamics (Mishra et al. 2011, Humplík et al. 2015, Lazarević et al. 2021). Although widely applied in crop species (Zhou et al. 2018, Cheong et al. 2019, Enders et al. 2019, Lazarević et al. 2021, Lazarević et al. 2022), these tools remain underused in extremophiles such as resurrection plants.

Despite their remarkable tolerance to desiccation and low temperatures, the physiological mechanisms underlying responses to cold, freezing and subsequent recovery in *Ramonda* spp. remain insufficiently understood. Cold and freezing stress affect plant physiology through partly overlapping but distinct mechanisms. Chilling temperatures can impair photosynthetic performance by inducing photoinhibition, restricting electron transport and reducing CO_2_ assimilation efficiency. In contrast, freezing stress additionally involves ice formation, which usually begins in extracellular spaces and lowers tissue water potential, thereby promoting cellular dehydration and membrane-associated stress (Thomashow 1998). Consequently, freezing injury often reflects the combined effects of low temperature and dehydration. This interaction is particularly relevant in resurrection plants, whose survival depends on efficient tolerance of cellular water loss and rapid recovery after rehydration. Because freezing can simultaneously impose low-temperature and dehydration stress, we aimed to evaluate the integrated physiological responses of *Ramonda* species to sequential nonfreezing cold and freezing stress, followed by rehydration and recovery. Therefore, this study compares the responses of *R. serbica* and *R. nathaliae* to low positive and subzero temperatures and examines their recovery following freezing-induced dehydration. This represents the first integrated phenotyping assessment of cold and freezing stress in these species. Our objective was to quantify temporal dynamics of key physiological traits during stress and recovery using chlorophyll fluorescence imaging, multispectral imaging and gas-exchange analysis, providing new insights into freezing tolerance in these resurrection plants.

## Materials and methods

### Plant material and growth conditions

Plants of *R. serbica* and *R. nathaliae* were collected from natural populations in Kosovo. *Ramonda nathaliae* was collected near Glloboçicë (UTM Zone 34N, WGS84: 515118 E, 4669685 N), whereas *R. serbica* was collected near Radavc (UTM Zone 34N, WGS84: 443216 E, 4731643 N) (Gashi et al. 2015). Plant material was collected under an official scientific research permit issued by the Ministry of Environment, Spatial Planning and Infrastructure of the Republic of Kosovo (Permit No. 2350/23, issued on 10 May 2023), in accordance with the Kosovo Law on Nature Protection (Law No. 03/L-233) and the conditions specified in the permit. Twenty mature individuals of each species were collected, and all individuals survived transport to the University of Zagreb, Faculty of Agriculture, and subsequent transplantation.

Twelve healthy, fully developed individuals of comparable size were selected for the phenotyping experiment, and the rest (8) were used for RWC measurement under equal experimental conditions. Each plant was transplanted into 1 L pots containing ProLine Container Coarse ‘Blueberries’ substrate (Klasmann-Deilmann GmbH, Geeste, Germany) and placed in a growth chamber at 20 °C, under a 16/8 h light/dark photoperiod and a photosynthetic photon flux density (PPFD) of 150 µmol m^−2^ s^−1^ provided by Valoya L35 lamps, NS12 spectrum (Valoya Oy, Helsinki, Finland). This PPFD was selected to reflect the shaded, low-light conditions of *Ramonda* understory habitats, in accordance with previous ex situ studies on these species (Gashi et al. 2013, 2025; Kastrati et al. 2025).

Both *Ramonda* species naturally grow under forest canopy in shaded, rocky habitats. In the studied populations, *R. nathaliae* was collected within the association *Ostryetum carpinifoliae*, whereas *R. serbica* originated from forest communities dominated by *Fraxinus ornus*. These habitats differ in microclimatic conditions, with *R. nathaliae* generally occurring in warmer, relatively drier environments, whereas *R. serbica* is frequently found in cooler, more humid sites (Gashi et al. 2013, Berisha et al. 2019, Kastrati et al. 2025). Both species are perennial, long-lived resurrection plants, and the individuals used in this study were mature adult rosettes collected in early autumn 2024, after completion of the reproductive phase. Detailed *in situ* measurements of photosynthetically active radiation (PAR), air temperature and relative humidity were not continuously recorded during sampling; therefore, natural growth conditions are described based on previously published ecological characterizations of the study sites (Gashi et al. 2013, Kastrati et al. 2025).

### Growth-chamber conditions and experimental phases

After a 10-day acclimation period (Phase I), plants were exposed to sequential cold and freezing treatments. Light conditions remained constant throughout the experiment (16/8 h light/dark and 150 µmol m^−2^ s^−1^ PPFD). The same photoperiod was maintained across all experimental phases, providing consistent light conditions while temperature was progressively reduced. This photoperiod was not intended to simulate the natural winter day length at the collection sites. Soil volumetric water content (VWC) was monitored daily (ThetaProbe ML2x; Delta-T Devices, Cambridge, UK) and maintained at 40% during Phases I–III. During freezing (Phases IV–V), substrate was allowed to freeze without irrigation; VWC was restored to 40% during recovery. A target VWC of 40% was chosen as a well-watered, nonlimiting moisture level for *Ramonda* plants, based on our previous experiments with similar peat-based substrates monitored by the same ThetaProbe sensor, where approximately 40% VWC consistently represented control, well-hydrated conditions and was used as the reference moisture level in drought studies (Lazarević et al. 2021, 2022, 2026).

Phase II comprised cold acclimation, during which the temperature was gradually reduced from 20 to 10 °C over 10 days at a rate of 1 °C day^−1^. This was followed by Phase III (cold stress), in which the temperature was further reduced from 10 to 0 °C over 10 days at the same rate of 1 °C day^−1^. During Phase IV (freezing stress), the temperature was lowered from 0 to −5 °C over 10 days at a rate of 1 °C every second day. In Phase V (freezing-induced desiccation), plants were maintained at −10 °C for 10 days. Finally, during Phase VI (recovery), plants were rehydrated and maintained at 20/15 °C (light/dark). The substrates were allowed to thaw and were subsequently watered to restore the VWC to 40%.

### Experimental design and measurements

The experiment followed a controlled repeated-measures design, with 12 plants per species monitored throughout the sequential cooling, freezing, and recovery phases (I–VI). Plants were randomly arranged within the growth chamber. Measurements were performed at the end of each experimental phase (I–V) and during recovery (Phase VI) at 1, 3, 6, 9, 12, 24, 48, 72, and 168 h (7 days). The measurements included chlorophyll fluorescence imaging, multispectral imaging, and gas exchange, with gas-exchange measurements omitted during the freezing phases, because tightly rolled leaves did not provide sufficient exposed and flattened leaf area for reliable measurements in the cuvette. Gas-exchange analysis, therefore, commenced 6 h after rehydration, when enough leaf surface had unfolded to ensure a stable seal in the cuvette. In contrast, chlorophyll fluorescence and multispectral imaging were performed during these phases, because these measurements were acquired from intact rosettes in a top view configuration. As also illustrated (Fig. 1), the first visible unfolding occurred in the central, younger leaves, providing sufficient optically accessible tissue even while the outer leaves remained rolled. An additional eight plants were grown under the same experimental conditions and were used for destructive RWC analyses.

**Figure 1:**
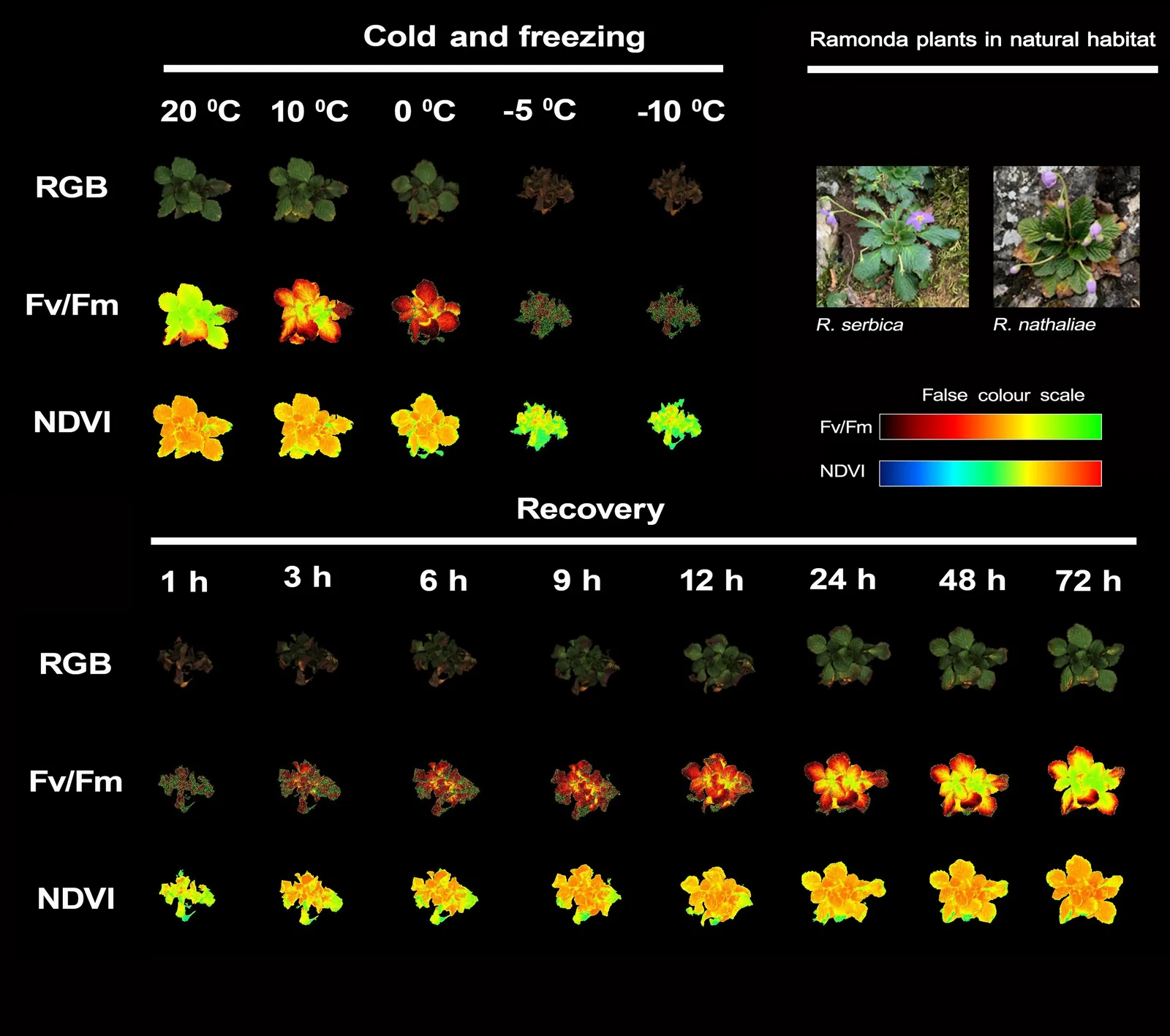
Representative RGB, maximum efficiency of PSII in the dark-adapted state (*F_v_*/*F_m_*), and normalized difference vegetation index (NDVI) images of *Ramonda nathaliae* during cold and freezing phases (20 to −10 °C) and subsequent recovery (1–72 h). The images show progressive changes in leaf morphology, PSII efficiency, and canopy greenness with decreasing temperature, followed by gradual restoration during recovery. Photographs of *R. serbica* and *R. nathaliae* in their natural habitats provide visual context for the study species.

#### Leaf RWC

Leaf RWC was determined using the gravimetric method of Weatherley (1950). Three leaves (one per plant) were sampled at each time point from plants not used for imaging or gas-exchange measurements. Fresh weight (FW) was recorded immediately. Leaves were then placed between moist filter paper, sealed in plastic bags, and kept at 4 °C for 24 h to reach full turgor. After blotting excess water, turgid weight (TW) was measured. Leaves were dried at 80 °C for 48 h to constant dry weight (DW). RWC was calculated as:


$$RWC(\text{\%})=\frac{FW-DW}{TW-DW}\times 100.$$

#### Chlorophyll fluorescence and multispectral imaging

Chlorophyll fluorescence, colour (RGB), and multispectral imaging were performed on 12 plants per species using the CropReporter™ system (PhenoVation B.V., Wageningen, Netherlands), positioned 30 cm above the leaves. The system enables noninvasive imaging of chlorophyll fluorescence and spectral reflectance, allowing spatially resolved assessment of photosynthetic performance and leaf optical properties. Lazarević et al. (2021) provide a detailed description of the system. Representative images from the cold, freezing, and recovery phases are shown (Fig. 1). The subsequent sections describe the chlorophyll fluorescence and multispectral imaging protocols.

### Chlorophyll fluorescence imaging

Chlorophyll fluorescence was measured using the optimized quenching protocol (dark-to-light induction; Brestic and Zivcak 2013). Plants were dark-adapted overnight, after which a saturating pulse (4500 µmol m^−2^ s^−1^, 800 ms) was applied to record *F*_0_ (10 µs) and *F_m_*. Four dark frames and 20 induction frames were acquired (integration time 200 ms).

Following a 15 s dark relaxation, actinic light (300 µmol m^−2^ s^−1^) was applied for 5 min to reach a light-adapted state. Steady-state fluorescence (*F_s_*) and maximum fluorescence of light-adapted leaves (*F_m_*′) were then recorded using the same saturating pulse. The same acquisition settings (four dark frames, 20 induction frames, 200 ms integration time) were used. After switching off the actinic light, minimal fluorescence in the light (*F*_0_′) was estimated under far-red illumination. All measured parameters were used to calculate the standard chlorophyll fluorescence traits (CFTs) (Table 1).

**Table 1:** List of calculated chlorophyll fluorescence, colour and multispectral parameters, including abbreviations, the equation for computation, and a reference if applicable.

Abbreviation	Trait	Equation	References
*F_v_/F_m_*	Maximum efficiency of PS II in the dark-adapted stage	*F_v_/F_m_* = (*F_m_* − *F*_0_)/*F_m_*	Kitajima and Butler (1975)
*F_q_*′/*F_m_*′	Effective quantum yield of PS II in the light-adapted stage	*F_q_*′/*F_m_*′ = (*F_m_*′ − *F_s_*)/*F_m_*′	Genty et al. (1989)
rETR	Relative electron transport rate	rETR = Fq′/Fm′ × PPFD × 0.5	Genty et al. (1989)
NPQ	Nonphotochemical quenching	NPQ = (*F_m_* − *F_m_*′)*/F_m_*′	Bilger and Björkman (1990)
qP	Coefficient of photochemical quenching	qP = (*F_m_*′ − *F_s_*)/(*F_m_*′ − *F*_0_′)	Schreiber et al. (1986)
qN	Coefficient of nonphotochemical quenching	qN = 1 − (*F_m_*′ − *F*_0_′)/(*F_m_* − *F*_0_)	Schreiber et al. (1986)
qL	Estimation of ‘open’ reaction centres based on a lake model	qL = [(*F_m_*′ − *F_s_*) × *F*_0_′]/[(*F_m_*′ − *F*_0_′) × *F_s_*]	Kramer et al. (2004)
ΦNO	Quantum yield of nonregulated nonphotochemical energy loss in PSII	ΦNO = 1/[NPQ + 1 + qL(*F_m_*/*F*_0_ − 1)]	Genty et al. (1996)
ΦNPQ	Quantum yield of regulated nonphotochemical energy loss in PSII	ΦNPQ = 1 − Fq′/Fm′ − ΦNO	Genty et al. (1996)
HUE	Hue (0–360°)	HUE = 60 × [0 + (RGreen − RBlue)/(max − min)] if max = RRed; 60 × [2 + (RBlue − RRed)/(max − min)] if max = RGreen; 60 × [4 + (RRed − RGreen)/(max − min)] if max = RBlue; 360 added if HUE < 0	—
SAT	Saturation (0–1)	SAT = (max (RRed, RGreen, RBlue)− min(RRed, RGreen, RBlue))/max if max > 0; SAT = 0 if max = 0	—
VAL	Value (0–1)	VAL= max (selected from RRed, RGreen, RBlue)	—
ARI	Anthocyanin index	ARI = (R550)^−1^ − (R710)^−1^	Gitelson et al. (2001)
CHI	Chlorophyll index	CHI = (R710)^−1^ − (R769)^−1^	Gitelson et al. (2003)
NDVI	Normalized difference vegetation index	NDVI = (RNIR − RRed)/(RNIR + RRed)	Rouse et al. (1974)
NPCI	Normalized pigments chlorophyll ratio index	NPCI = (RRed − RBlue)/(RRed + RBlue)	Peñuelas et al. (1995)
GLI	Green leaf index	GLI = (2 × RGreen − RRed − RBlue)/(2 × RGreen + RRed + RBlue)	Gobron et al. (2000)

### Multispectral imaging

Following the fluorescence protocol, an actinic light of 250 µmol m^−2^ s^−1^ was applied for ∼1–2 min to record RGB and multispectral reflectance under a light-adapted steady state, a moderate irradiance commonly used in similar phenotyping setups (Lazarević et al. 2021, 2022, 2026).

Spectral reflections in red (*R*_Red_—640 nm), green (*R*_Green_—550 nm), blue (*R*_Blue_—475 nm), specific green (*R*_SpcGrn_—510–590 nm), chlorophyll reflectance (*R*_Chl_—730 nm) near infra-red (*R*_NIR_—769 nm), and far-red (*R*_FarRed_—710 nm) were captured as whole leaf images. Again, DA software was used to calculate the colours of the collected images, multispectral reflectance, and vegetation indices (Table 1).

#### Gas-exchange analysis

Gas exchange was measured in the growth chamber during Phases I–III and VI (6–168 h) using a LI-6800 system (LI-COR Biosciences, Lincoln, Nebraska, USA) equipped with a 1 × 3 cm clear-top chamber (6800-12A). Prior to the start of the temperature treatments, light-response curves were recorded once on three plants per species to determine a non-limiting irradiance for subsequent gas-exchange measurements. During these light-response measurements, the cuvette conditions were kept constant, with a reference CO_2_ concentration of 400 µmol mol^−1^, a flow rate of 600 µmol s^−1^, a chamber temperature of 20 °C, and a relative humidity of 55%, while only the incident PPFD was varied stepwise.

Net assimilation rate (*A*) was recorded across a series of increasing PPFD steps, and 300 µmol m^−2 ^s^−1^ (90:10 red:blue, 6800-02 light source) was selected as an operational saturating irradiance based on the A–PPFD response, i.e. the point at which A approached a stable plateau for both species, consistent with common empirical approaches to light-saturation determination in C_3_ plants (e.g. Medrano et al. 2002). Thereafter, all gas-exchange measurements during Phases I–III and VI were performed at 300 µmol m^−2 ^s^−1^ under the same cuvette settings.

During the cold phase, measurements were taken on all plants (*n* = 12), while during recovery they were performed on five plants per species due to the higher sampling frequency. Each measurement was conducted inside the growth chamber at the chamber’s programmed temperature (20, 10, or 0 °C in Phases I–III, and 20 °C during recovery). Although air temperature in the cuvette matched these set values, leaf temperature was consistently 0.5–1.0 °C higher. The reference CO_2_ concentration was 400 µmol mol^−1^, the flow rate 600 µmol s^−1^, and relative humidity was maintained at 55%, except at 0 °C, where it was reduced to 30% to prevent condensation. Data were logged once CO_2_ and H_2_O signals stabilized (ΔCO_2_ < 0.5 µmol mol^−1^ min^−1^; ΔH_2_O < 0.15 mmol mol^−1^ min^−1^). Gas analysers were matched before each measurement, and a 15-s averaging interval was used. From each gas-exchange measurement, net assimilation rate (*A*), stomatal conductance (*g*_sw_), transpiration (*E*) and intercellular CO_2_ concentration (Ci) were obtained directly from the LI-6800 output. Intrinsic water-use efficiency (WUEi) was calculated as *A*/*g*_sw_, and the *A*/Ci ratio was derived from the corresponding A and Ci values.

### Data analysis

All analyses were performed in JMP Pro 18 (SAS Institute Inc., Cary, NC, USA). Plant traits were grouped into CFT, multispectral trait (MST) and gas-exchange trait (GAST).

#### Dimensionality reduction and variable selection

Due to strong multicollinearity, variable clustering was applied to CFT and MST datasets to reduce dimensionality and identify nonredundant representative traits. Clusters were formed based on intercorrelation structure, and one trait per cluster was selected according to the lowest 1 − *R*^2^ ratio or physiological relevance. Because the gas-exchange traits represented a relatively small set of physiologically distinct variables, they were analysed individually. Complete clustering results and the selected representative traits are provided in Supplementary Tables S1 (CFT) and S2 (MST).

#### Regression model selection for trait dynamics

We modelled the temporal dynamics of representative traits separately for each experimental phase. Temperature was used as the predictor during the cold and freezing phases, whereas time was used during the recovery phase. Candidate models included linear, quadratic, exponential and logistic functions. Model performance was evaluated using AICc (with AICc weight), BIC, RMSE, MSE and *R*^2^. For each trait and phase, the model with the lowest AICc and strongest biological plausibility was selected. Detailed model comparisons are provided in Supplementary Tables S3 (cold/freezing) and S4 (recovery).

#### Linear mixed-effects modelling

Linear mixed-effects models (LMMs) were used to test the effects of species and treatment while accounting for repeated measurements on the same individual plants during the cold/freezing and recovery phases. For cold/freezing datasets, species (*R. nathaliae* and *R. serbica*), temperature treatment (20, 10, 0, −5, and −10 °C), and their interaction were fitted as fixed effects, while individual plant identity was included as a random factor to account for repeated measurements. Plant identities were unique across species (*R. nathaliae*: Subjects 1–12; *R. serbica*: Subjects 13–24), and the same Subject ID was retained for each individual across all repeated measurements. Thus, each individual plant was uniquely associated with a single species and plant identity was inherently nested within species. For recovery datasets, species, recovery time (depending on the dataset: 1–168 h or 6–168 h for gas exchange), and their interaction were treated as fixed effects, with individual plant identity included as the random factor following the same subject structure. Separate models were fitted for RWC, representative CFT (*F_v_*/*F_m_*, *F_q_*′/*F_m_*′, NPQ), MST (*R*_SpcGrn_, NDVI), and gas-exchange parameters (*A*, *E*, *g*_sw_, Ci, WUEi, and A/Ci).

The significance of fixed effects was evaluated using analysis of variance (ANOVA), with degrees of freedom, *F*-values, and *P*-values reported for each main effect and interaction. Estimated marginal means (EMMs) were calculated from the fitted models, and Tukey-adjusted pairwise comparisons were used to compare treatment levels where appropriate. Differences among treatment levels within each species were summarized using compact letter displays (CLDs), where means sharing the same letter were not significantly different at *P* < 0.05 (Supplementary Tables S5A for the cold and freezing phases and S6A for recovery). To compare the two species at individual temperature or recovery time levels, we performed pairwise contrasts of EMMs (*R. serbica–R. nathaliae*) within each level of the respective treatment factor. These contrasts were used as follow-up comparisons to the LMM analyses and are reported in Supplementary Tables S5B for the cold and freezing phases and S6B for recovery.

## Results

### Cluster variables for variable selection results

Variable clustering effectively reduced dimensionality within CFT and MST datasets by grouping highly correlated traits into functionally coherent clusters (Supplementary Tables S1–S2). For CFT, 14 variables were grouped into three clusters, explaining 88.7% of the total variance. These clusters represented: (1) maximum efficiency of PSII in the dark-adapted state (*F_v_*/*F_m_* cluster, 57.8%), (2) effective quantum yield of PSII in the light-adapted state (*F_q_*′/*F_m_*′ cluster, 18.3%), and (3) nonphotochemical quenching (NPQ cluster, 12.6%). Although *F*_0_ had the lowest 1 − *R*^2^ ratio within Cluster 1, *F_v_*/*F_m_* was selected as the representative trait because of its clearer physiological relevance as an indicator of maximum PSII efficiency (Supplementary Table S1). For MST, 14 traits formed two clusters explaining 85.8% of total variance: (1) specific green reflectance (*R*_SpcGrn_ cluster, 49.8%) and (2) normalized difference vegetation index (NDVI cluster, 36.0%). Other RGB-derived colour parameters, as well as other vegetation indices, showed high redundancy with *R*_SpcGrn_ and NDVI and were therefore not retained as separate representative traits in subsequent analyses.

High within-cluster cohesion (*R*^2^ > 0.75) and low cross-cluster correlations confirmed strong trait redundancy within each group. Based on both statistical representativeness and physiological relevance, *F_v_*/*F_m_*, *F_q_*′/*F_m_*′, NPQ, *R*_SpcGrn_, and NDVI were selected as representative traits for subsequent regression modelling.

### Leaf RWC dynamics in *R. serbica* and *R. nathaliae* during cold, freezing and recovery phases

Leaf RWC showed clear and fully reversible changes across cold, freezing, and recovery phases (Fig. 2). The logistic 3-P model best described both dehydration (*R*^2^ = 0.96) and rehydration (*R*^2^ = 0.98) dynamics (Supplementary Tables S3 and S4).

**Figure 2:**
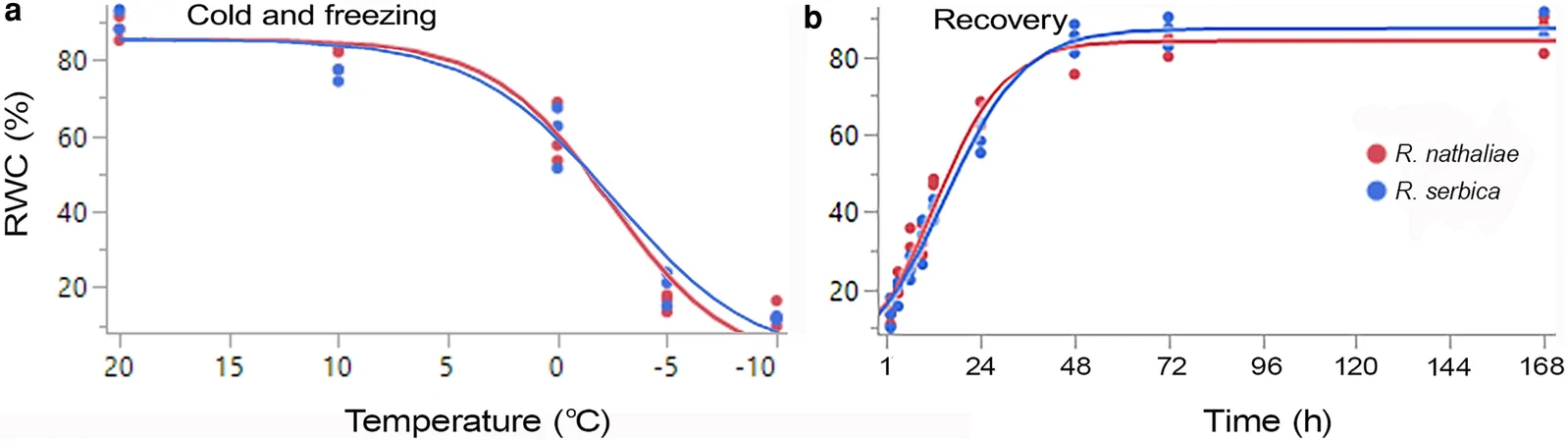
Relative water content (RWC) dynamics in *Ramonda* species during cold and freezing (a) and recovery (b) phases.

During cold and freezing (Fig. 2a), both species exhibited a gradual decline in RWC followed by a sharper drop near the freezing point. *Ramonda nathaliae* had a fitted growth-rate parameter of 0.37 and reached its inflection point earlier (−1.95 °C) compared with a growth-rate parameter of 0.31 and an inflection point of −2.20 °C in *R. serbica*. Despite these differences in fitted model parameters, curve shapes did not differ significantly, indicating broadly similar dehydration patterns. RWC decreased from 88.34% to 12.78% in *R. nathaliae* and from 91.57% to 11.94% in *R. serbica* between 20 and −10 °C. The LMM revealed a strong effect of temperature, whereas species and the temperature × species interaction were not significant (Table 2). Tukey-adjusted pairwise comparisons of EMMs among temperature levels within each species showed that RWC remained stable between 20 and 10 °C in *R. nathaliae* but declined stepwise at lower temperatures, whereas in *R. serbica* successive temperature reductions generally resulted in significant decreases in RWC. However, pairwise contrasts of EMMs between species at individual temperature levels revealed no significant interspecific differences (Supplementary Table S5B).

**Table 2:** Effects of treatment, species and their interaction on relative water content (RWC) during cold/freezing and recovery phases in *Ramonda nathaliae* and *R. serbica*.

Source of variation	Cold/freezing phase			Recovery phase		
	df	*F*	*P*	df	*F*	*P*
Treatment^a^	4	246.16	<0.001	8	140.82	<0.001
Species	1	0.21	0.376	1	0.15	0.703
Treatment × Species	4	0.87	0.268	8	1.50	0.191

^a^For cold/freezing, treatment refers to temperature level; for recovery, treatment refers to time after rehydration.

During recovery (Fig. 2b), both species rehydrated rapidly, again fitting the logistic model (*R*^2^ = 0.98). Growth rates were comparable (0.12 in *R. nathaliae*; 0.10 in *R. serbica*), with fitted inflection points of 12.1 h in *R. nathaliae* and 15.1 h in *R. serbica*. The LMM revealed a strong effect of recovery time, whereas species and the time × species interaction were not significant (Table 2). Pairwise comparisons at individual sampling times also detected no significant interspecific differences in RWC (Supplementary Table S6B). Within each species, RWC increased from low values shortly after rehydration to near-control levels by 48–168 h, indicating that most rehydration occurred within the first 24–48 h, after which both species stabilized near full hydration (Fig. 2b; Supplementary Table S6A). Overall, both *Ramonda* species exhibited the characteristic desiccation–rehydration cycle of resurrection plants, with similar RWC responses and no statistically supported species differences during either dehydration or recovery.

### Chlorophyll fluorescence dynamics in *R. serbica* and *R. nathaliae* during cold, freezing, and recovery phases

Representative RGB, *F_v_*/*F_m_* and NDVI images of *R. nathaliae* during cooling, freezing and subsequent recovery illustrate the progressive decline and subsequent restoration of PSII efficiency and canopy greenness at the whole-rosette level (Fig. 1).

The *F_v_*/*F_m_* declined progressively with decreasing temperature in both species (Fig. 3a). At 20 °C, *F_v_*/*F_m_* was 0.67 ± 0.021 in *R. nathaliae* and 0.65 ± 0.013 in *R. serbica*. As temperature decreased, *F_v_*/*F_m_* declined to 0.44 and 0.47 at 10 °C, and reached minima of 0.28–0.3 in *R. nathaliae* and 0.27–0.28 in *R. serbica* at −5 and −10 °C. A quadratic model best described this trend (*R*^2^ = 0.89; Supplementary Table S3), indicating gradual PSII downregulation during cooling and freezing. Although *R. serbica* maintained slightly higher *F_v_*/*F_m_* at intermediate temperatures (e.g. 10 °C), the overall temperature response was similar in both species, as indicated by the nonsignificant temperature × species interaction, despite a significant overall species effect (Table 3). Pairwise species comparisons at individual temperature levels did not reveal consistent interspecific differences (Supplementary Table S5B). During recovery, *F_v_*/*F_m_* increased rapidly in both species, following a logistic 3-P model (*R*^2^ = 0.93; Fig. 3b). *Ramonda serbica* showed a faster recovery rate (0.032 vs 0.023) but a later inflection point (11.5 h vs 8.0 h), suggesting slightly slower initiation but stronger late-stage stabilization. Both species reached similar asymptotic values (∼0.68), close to pre-stress levels, confirming substantial PSII recovery. Recovery kinetics differed significantly at the curve-fit level, but the LMM revealed a strong effect of recovery time, whereas species and the time × species interaction were not significant (Table 3), indicating that *F_v_*/*F_m_* recovery was primarily driven by recovery duration rather than species identity (Supplementary Tables S5B and S6B).

**Figure 3:**
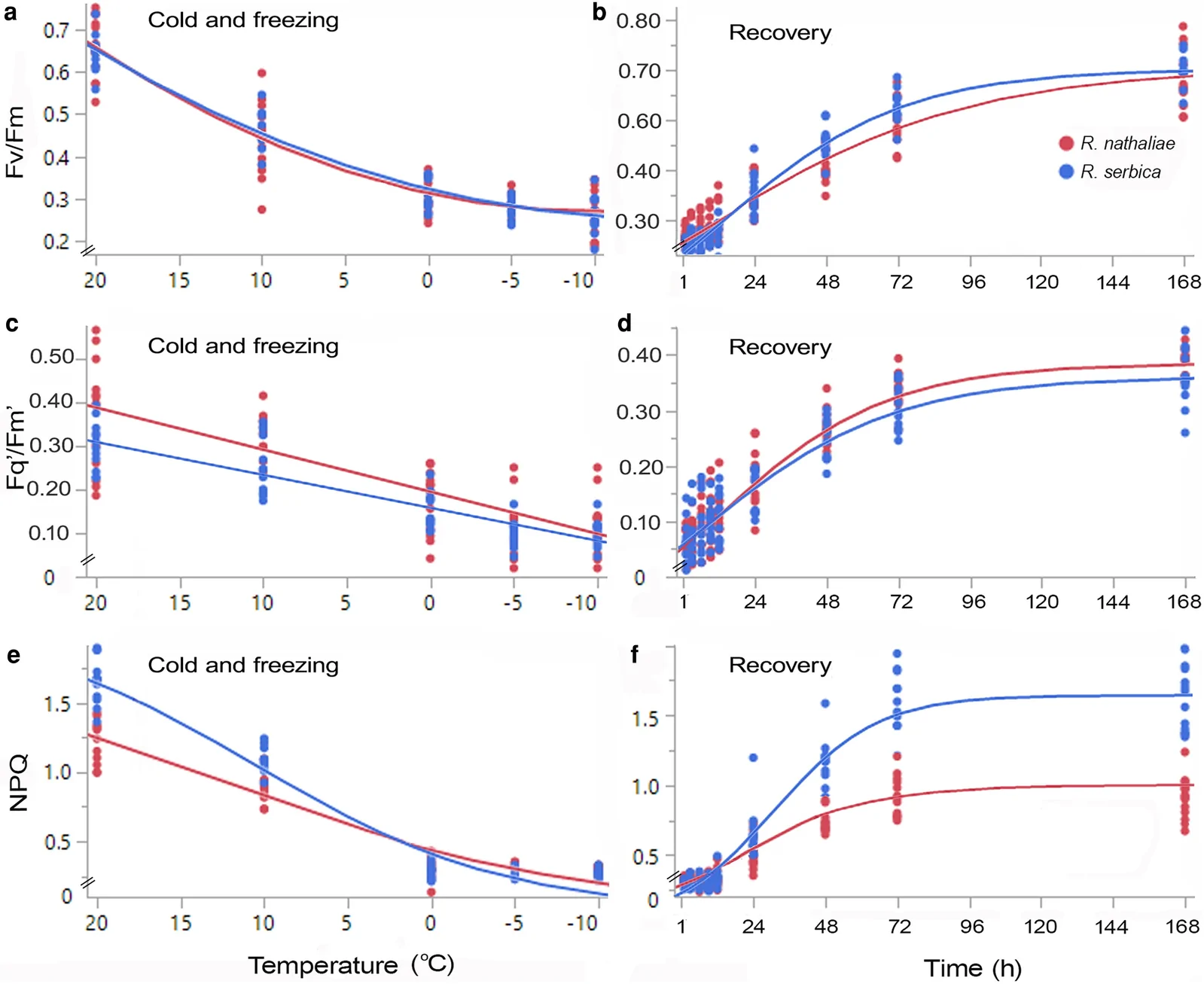
Dynamics of chlorophyll fluorescence parameters in *Ramonda* species during cold and freezing (a, c, e) and recovery phases (b, d, f). (a, b) Maximum efficiency of PSII in the dark-adapted state (*F_v_/F_m_*), (c, d) effective quantum yield of PSII in the light-adapted state (*F_q_*′/*F_m_*′), and (e, f) nonphotochemical quenching (NPQ).

**Table 3:** Effects of treatment, species and their interaction on chlorophyll fluorescence traits during the cold/freezing and recovery phases in *Ramonda nathaliae* and *R. serbica*.

Source of variation	df	Cold/freezing phase						df	Recovery phase					
		*F_v_/F_m_*		*F_q_*′/*F_m_*′		NPQ			*F_v_/F_m_*		*F_q_*′/*F_m_*′		NPQ	
		*F*	*P*	*F*	*P*	*F*	*P*		*F*	*P*	*F*	*P*	*F*	*P*
Treatment^a^	4	295.73	<0.001	68.50	<0.001	321.4	<0.001	8	462.6	<0.001	89.3	<0.001	331.7	<0.001
Species	1	7.17	0.014	0.21	0.655	85.56	<0.001	1	2.25	0.148	0.94	0.344	7.71	0.011
Treatment × Species	4	0.05	0.996	1.55	0.194	28.61	<0.001	8	1.22	0.287	1.46	0.176	2.95	0.004

^a^For cold/freezing, treatment refers to temperature level; for recovery, treatment refers to time after rehydration. *F_v_/F_m_*, maximum quantum efficiency of PSII in the dark-adapted state; *F_q_*′/*F_m_*′, effective quantum yield of PSII in the light-adapted state; NPQ, nonphotochemical quenching.

The effective quantum yield of PSII in the light-adapted state (*F_q_*′/*F_m_*′) also changed markedly during the experiment. During cold/freezing, *F_q_*′/*F_m_*′ declined significantly with decreasing temperature (Table 3) and was adequately described by linear fits (Supplementary Table S3), whereas during recovery, it increased progressively and was best described by a logistic 3-P model (*R*^2^ = 0.88; Fig. 3d). *Ramonda serbica* initiated recovery earlier (inflection at 4.6 h vs. 7.6 h), whereas *R. nathaliae* showed a steeper increase after rehydration onset, and curve kinetics differed significantly. Nevertheless, both species converged to very similar final *F_q_*′/*F_m_*′ values (0.38 vs. 0.37). The LMM showed a significant effect of recovery time, while species and the time × species interaction were not significant (Table 3), indicating a common temporal recovery pattern in both species (Supplementary Tables S5B and S6B). NPQ declined with decreasing temperature and then rose sharply during recovery (*R*^2^ = 0.93; Fig. 3f). During the cold/freezing phase, NPQ was significantly affected by temperature, species and their interaction (Table 3), indicating that the magnitude of the temperature response differed between species. *Ramonda serbica* recovered more rapidly and reached substantially higher asymptotic NPQ values than *R. nathaliae*. During recovery, NPQ was significantly affected by time, species and the time × species interaction (Table 3), indicating species-specific recovery dynamics. Species differences became more pronounced over time; from 24 h onward, *R. serbica* consistently exhibited higher NPQ values than *R. nathaliae*, reaching maxima at 72–168 h (Supplementary Tables S5A, S5B, S6A and S6B).

Overall, chlorophyll fluorescence parameters during recovery were predominantly influenced by time, reflecting progressive restoration of PSII functionality after freezing stress, whereas NPQ exhibited clear species-specific differences. Taken together, these patterns suggest that *R. serbica* exhibited a stronger NPQ-based photoprotective energy dissipation response during recovery, while both species restored photochemical efficiency (*F_v_*/*F_m_* and *F_q_*′/*F_m_*′) in a broadly similar manner.

### Multispectral trait dynamics in *R. serbica* and *R. nathaliae* during cold, freezing, and recovery phases

During the cold and freezing phase, NDVI changes were best described by a quadratic model (*R*^2^ = 0.83), indicating a nonlinear decline in canopy greenness with decreasing temperature (Supplementary Table S3; Fig. 4a). Both *R. serbica* and *R. nathaliae* maintained relatively stable NDVI under moderate chilling, followed by a gradual decrease near and below 0 °C. NDVI declined from 0.783 ± 0.009 to 0.540 ± 0.012 in *R. nathaliae* and from 0.786 ± 0.004 to 0.556 ± 0.004 in *R. serbica*, with similar quadratic coefficients in both species, indicating comparable temperature responses. Specific green reflectance (*R*_SpcGrn_) during cold and freezing was best described by a logistic 3-P model (*R*^2^ = 0.82; Fig. 4c). Both species showed early reflectance shifts, with inflection points near the freezing threshold (−1.26 °C for *R. nathaliae*, −0.70 °C for *R. serbica*), and values decreased from 2741 ± 109 to 606 ± 23 in *R. nathaliae* and from 2788 ± 34 to 670 ± 24 in *R. serbica*. Despite pronounced temperature effects, the LMM detected an overall species effect for both NDVI and *R*_SpcGrn_, whereas the temperature × species interactions were not significant (Table 4). However, sliced species comparisons revealed no significant interspecific differences in either trait at individual temperatures (Supplementary Table S5B). During recovery, NDVI followed a logistic 3-P model (*R*^2^ = 0.81; Fig. 4b). Both species showed a rapid initial increase after rewarming, with *R. nathaliae* displaying a slightly higher growth rate (0.07 vs. 0.06). Inflection points just before the first measurement suggest that NDVI recovery had already begun during thawing. Final asymptotic values were similar (0.75 vs. 0.73), and species trajectories did not differ significantly according to the curve-comparison test. The LMM confirmed a strong effect of recovery time, whereas species and time × species were not significant (Table 4). Pairwise comparisons at individual time points also detected no significant interspecific differences (Supplementary Table S5B). In both species, NDVI increased from low initial values at 1–12 h to intermediate values at 24 h, reaching maximal levels from 48 h onward, indicating rapid restoration of leaf greenness (Supplementary Table S6A).

**Figure 4:**
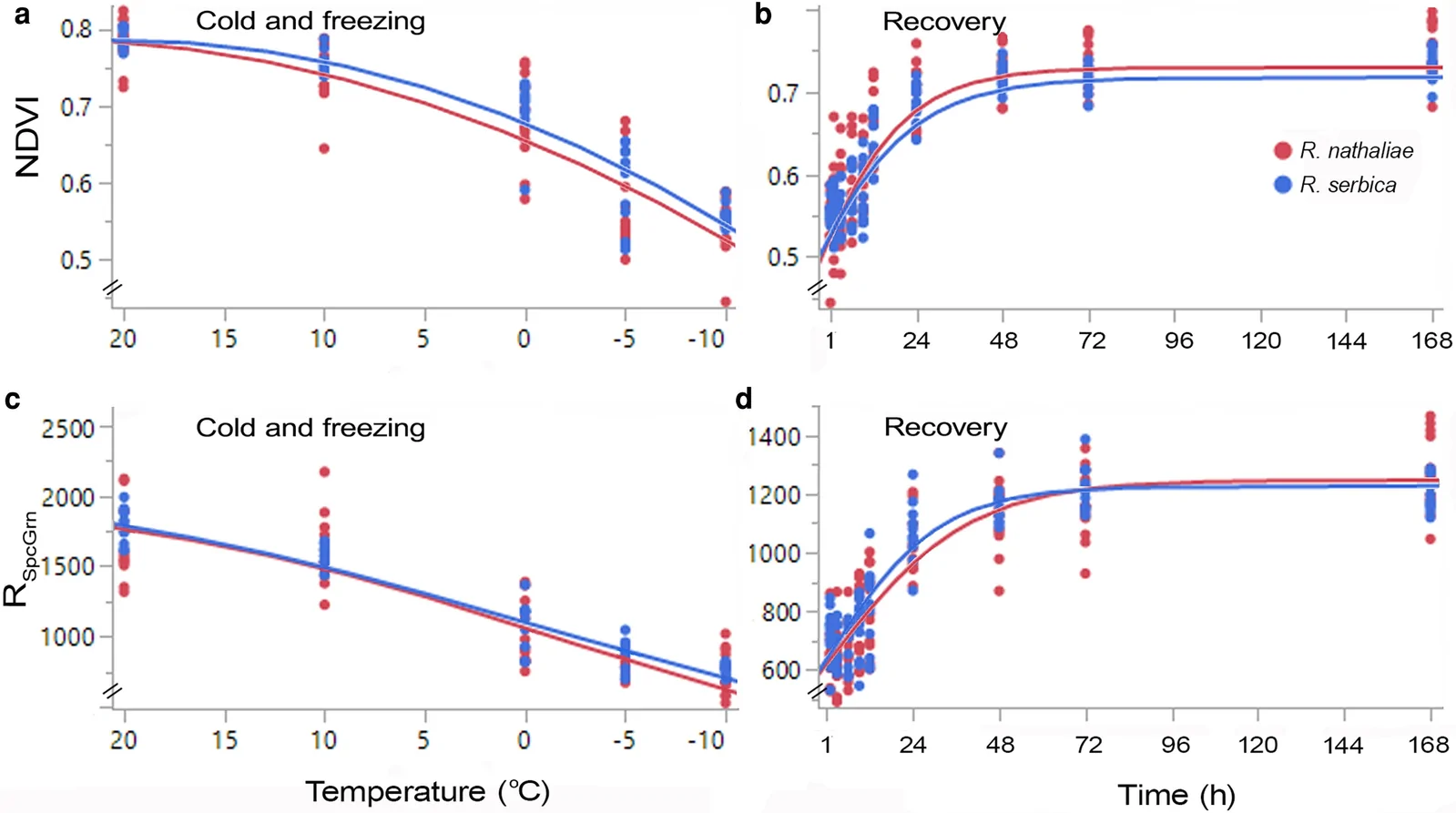
Dynamics of multispectral parameters in *Ramonda* species during cold and freezing (a, c) and recovery phases (b, d). (a, b) Normalized difference vegetation index (NDVI) and (c, d) specific green reflectance (*R*_SpcGrn_).

**Table 4:** Effects of treatment, species and their interaction on multispectral traits during the cold/freezing and recovery phases in *Ramonda nathaliae* and *R. serbica*.

Source of variation	df	Cold/freezing phase				df	Recovery phase			
		RSpcGrn		NDVI			RSpcGrn		NDVI	
		*F*	*P*	*F*	*P*		*F*	*P*	*F*	*P*
Treatment^a^	4	657.74	<0.001	211.85	<0.001	8	236.97	<0.001	237.07	<0.001
Species	1	10.33	0.004	5.68	0.026	1	1.95	0.176	0.64	0.434
Treatment × Species	4	1.14	0.345	0.95	0.437	8	1.35	0.223	1.08	0.381

^a^For cold/freezing, treatment refers to temperature level; for recovery, treatment refers to time after rehydration. RSpcGrn, specific green reflectance; NDVI, normalized difference vegetation index.

*R*_SpcGrn_ during recovery again followed a logistic 3-P pattern (*R*^2^ = 0.82; Fig. 4d). Both species exhibited gradual increases in reflectance, with *R. serbica* showing a slightly higher growth rate (0.061 vs 0.056). Inflection points again preceded the first measurement, indicating that pigment and structural restoration began during thawing. Although *R. serbica* reached marginally higher asymptotic values (1239.7 vs. 1215.8), trajectories did not differ significantly according to the curve-comparison test, and the LMM showed a significant effect of recovery time whereas species and the recovery time × species interaction were not significant (Table 4; Supplementary Table S6B). Within both species, *R*_SpcGrn_ increased steadily from low values at 1 h to near-maximal values from 48 h onward but remained below initial control levels (Supplementary Table S6A).

Overall, multispectral analyses showed that both *Ramonda* species exhibited highly similar spectral reflectance responses to freezing stress and subsequent rehydration. NDVI returned to near pre-stress values in both species, indicating effective recovery of leaf greenness, whereas *R*_SpcGrn_ recovered more gradually and remained slightly depressed, suggesting that the spectral properties captured by *R*_SpcGrn_ recovered more slowly than those reflected by NDVI.

### Gas-exchange dynamics in *R. serbica* and *R. nathaliae* during cold and recovery phases

Gas-exchange measurements were conducted only during the cold and recovery phases, as leaf rolling and technical limitations prevented measurements during freezing (−5 and −10 °C) and during the first 1–3 h of recovery. Linear mixed-effects models (LMMs) were used in both phases to test the effects of treatment (temperature or recovery time), species, and their interaction on gas-exchange parameters. In addition, the greater temporal resolution during recovery enabled regression modelling to characterize recovery kinetics. During cooling, temperature significantly affected net assimilation rate (*A*), intercellular CO_2_ concentration (Ci), stomatal conductance (*g*_sw_), transpiration rate (*E*), and intrinsic water-use efficiency (WUEi), whereas the A/Ci ratio remained unchanged (Table 5). Species effects were significant for *A*, *g*_sw_, *E*, and *A*/Ci, but not for Ci or WUEi. No significant temperature × species interactions were detected for any parameter (Table 5), indicating similar temperature-response patterns in both species. Across temperatures, *R. serbica* generally maintained higher *A*, *g*_sw_, and *E* values than *R. nathaliae* (Fig. 5), suggesting greater physiological activity under chilling conditions.

**Figure 5:**
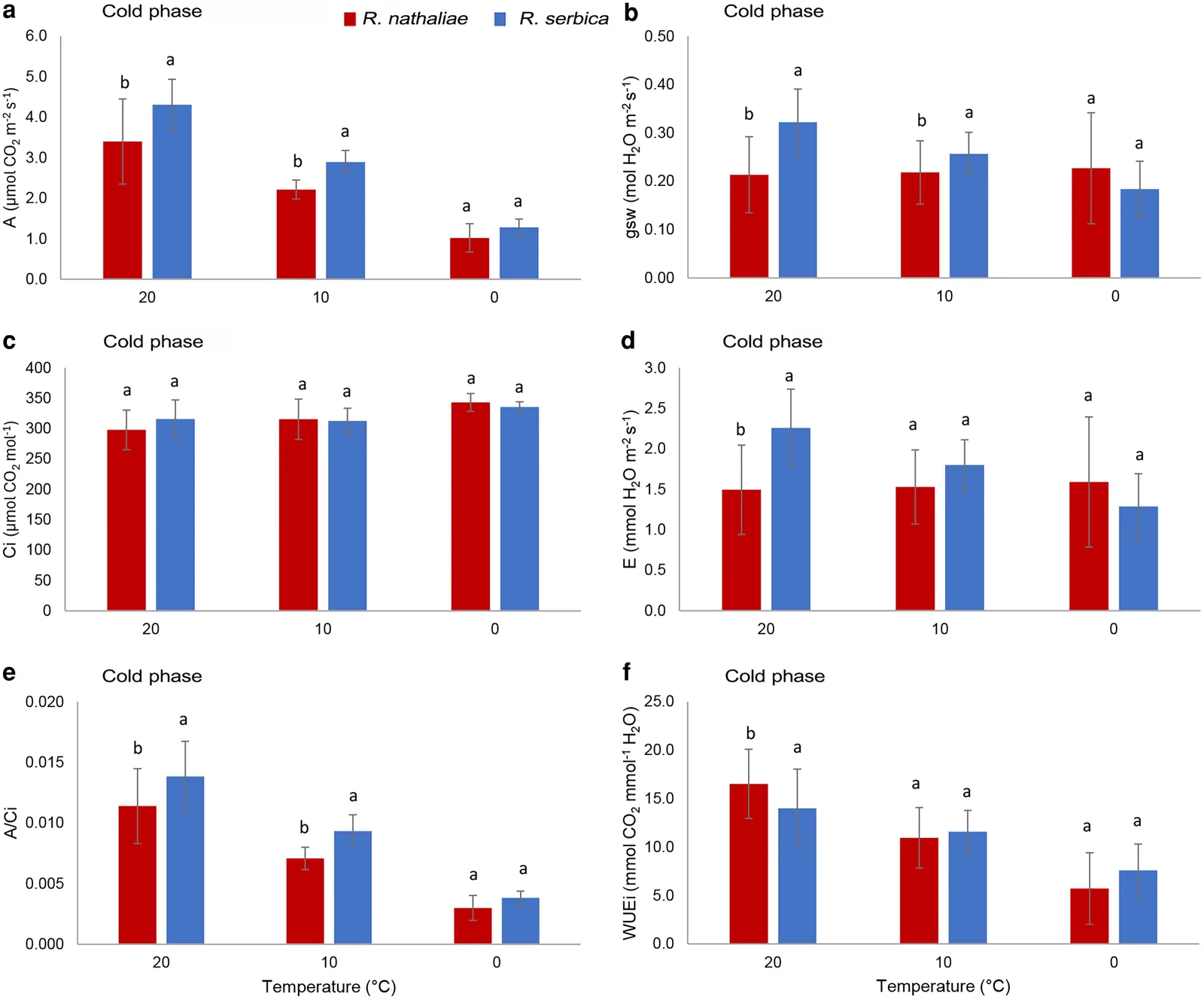
Temperature-dependent responses of leaf gas-exchange traits in *R. nathaliae* and *R. serbica* during cooling from 20 to 0 °C: (a) Net CO_2_ assimilation rate (a), (b) stomatal conductance to water vapour (gsw), (c) intercellular CO_2_ concentration (Ci), (d) transpiration rate (e), (e) A/Ci ratio, and (f) intrinsic water-use efficiency (WUEi). Different lowercase letters indicate significant differences among temperature levels within each species based on Tukey-adjusted pairwise comparisons of estimated marginal means (*P* < 0.05).

**Table 5:** Effects of treatment, species and their interaction on gas-exchange traits during the cold and recovery phases in *Ramonda nathaliae* and *R. serbica*.

Source of variation	df	Cold phase											
		*A*		Ci		*g*_sw_		E		WUEi		A/Ci	
		*F*	*P*	*F*	*P*	*F*	*P*	*F*	*P*	*F*	*P*	*F*	*P*
Temperature (T)	2	187.61	<0.001	8.86	0.001	13.94	<0.001	13.61	<0.001	20.69	<0.001	0.03	0.972
Species (S)	1	9.36	0.003	0.22	0.643	14.96	<0.001	12.10	0.001	0.05	0.817	11.20	0.002
T × S	2	1.24	0.296	1.65	0.201	1.84	0.169	1.73	0.187	0.39	0.680	0.80	0.453
		**Recovery phase**											
Time (T)	6	21.56	<0.001	43.10	<0.001	39.80	<0.001	39.80	<0.001	33.17	<0.001	23.79	<0.001
Species (S)	1	2.05	0.179	28.75	0.003	3.19	0.108	2.84	0.126	1.67	0.229	2.38	0.157
T × S	6	2.49	0.039	2.84	0.021	12.66	<0.001	12.66	<0.001	4.32	0.002	2.18	0.066

*A*, net CO_2_ assimilation rate; Ci, intercellular CO_2_ concentration; gsw, stomatal conductance; E, transpiration rate; WUEi, intrinsic water-use efficiency; *A*/Ci, apparent carboxylation efficiency. For the cold phase, *T* refers to temperature; for recovery, *T* refers to time after rehydration.

During recovery following freezing-induced desiccation, all gas-exchange traits were strongly affected by time (Table 5), indicating progressive changes of photosynthetic function after rehydration. Net assimilation rate increased steadily in both species and was best described by a linear model (*R*^2^ = 0.74) (Supplementary Table S4; Fig. 6a). Values rose from 0.59 µmol CO_2_ m^−2^ s^−1^ in *R. nathaliae* and 0.68 µmol CO_2_ m^−2^ s^−1^ in *R. serbica* at 6 h to 3.51 and 4.02 µmol CO_2_ m^−2^ s^−1^, respectively, at 168 h. Although the overall species effect was not significant, the significant time × species interaction (Table 5) indicates different recovery trajectories, with *R. serbica* showing a stronger late-stage increase (Supplementary Table S6A).

**Figure 6:**
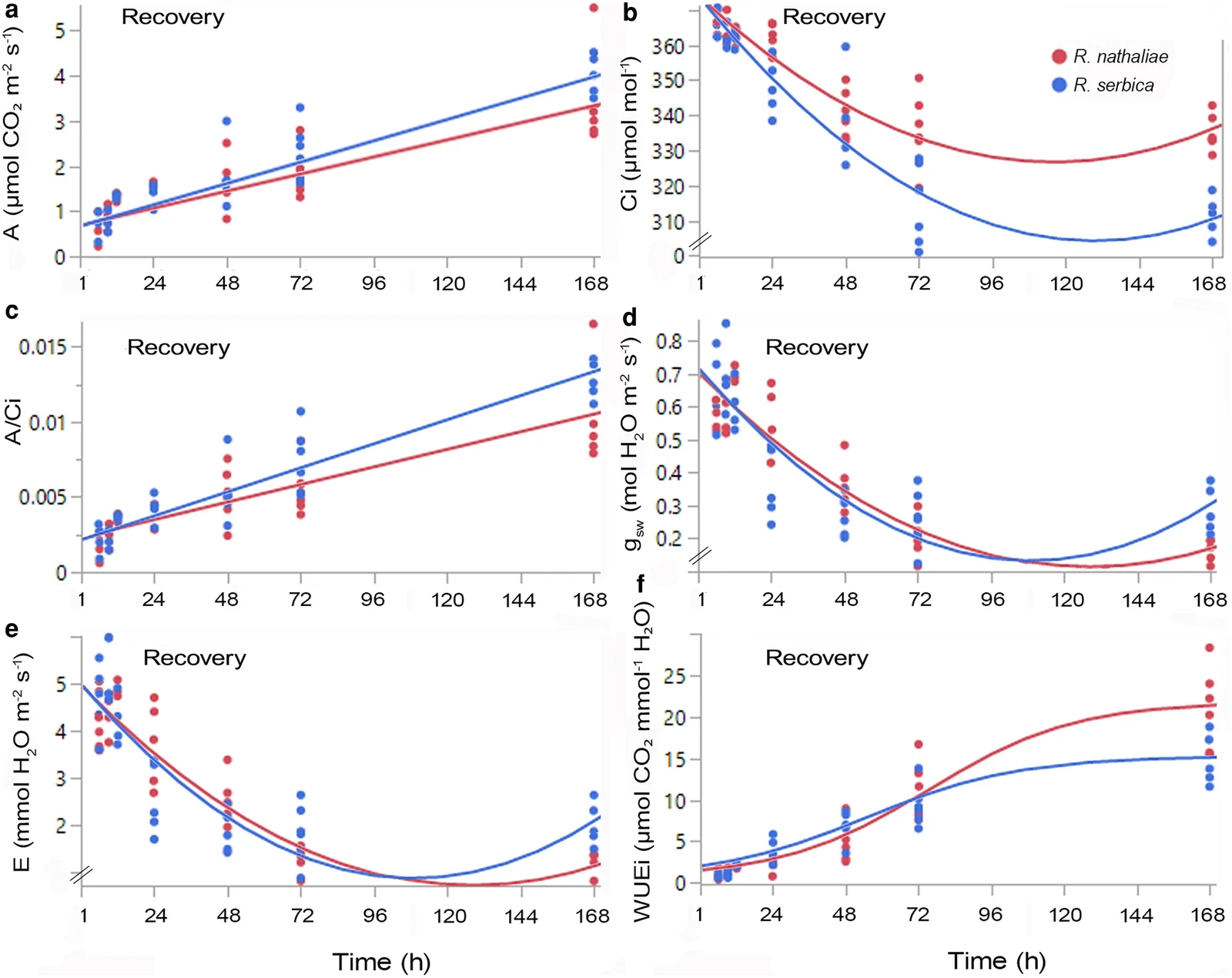
Dynamics of gas-exchange parameters in *Ramonda* species during recovery: (a) net CO_2_ assimilation rate (a), (b) intercellular CO_2_ concentration (CI), (c) A/Ci ratio, (d) stomatal conductance to water vapour (gsw), (e) transpiration rate (e), and (f) intrinsic water-use efficiency (WUEi).

Intercellular CO_2_ concentration (Ci) followed a quadratic recovery pattern (*R*^2^ = 0.85) (Supplementary Table S4; Fig. 6b), remaining high at 6–12 h (362–368 µmol mol^−1^) before declining to 335.59 µmol mol^−1^ in *R. nathaliae* and 310.36 µmol mol^−1^ in *R. serbica* at 168 h. Both recovery time and species effects were significant, as was their interaction (Table 5), indicating a greater late-stage decline in Ci in *R. serbica* during later recovery.

Stomatal conductance (*g*_sw_) and transpiration (*E*) were both best described by quadratic models during recovery (*R*^2^ = 0.77 for both; Supplementary Table S4; Fig. 6d and 6e). In both species, these traits remained relatively high during early recovery (6–24 h) and then declined towards later stages. In *R. nathaliae*, *g*_sw_ decreased from 0.55 to 0.17 mol H_2_O m^−2^ s^−1^ and *E* from 3.75 to 1.19 mmol H_2_O m^−2^ s^−1^ between 6 and 168 h, whereas in *R. serbica g*_sw_ declined from 0.68 to 0.29 mol H_2_O m^−2^ s^−1^ and *E* from 4.76 to 2.00 mmol H_2_O m^−2^ s^−1^. Time effects were highly significant for both variables, and significant time × species interactions were detected for both traits (Table 5), indicating distinct temporal response patterns between species. Species effects were not significant for *g*_sw_ or *E* (Table 5), although *R. serbica* generally maintained higher values during later recovery (Supplementary Table S6A; Fig. 6d and 6e).

Intrinsic water-use efficiency (WUEi) showed a strongly nonlinear increase during recovery and was best captured by a logistic 3-P model (*R*^2^ = 0.84; Supplementary Table S4; Fig. 6f). WUEi increased from approximately 1.0 at 6 h to 20.86 in *R. nathaliae* and 14.72 in *R. serbica* at 168 h. Time strongly affected WUEi, whereas the species main effect was not significant; however, the significant time × species interaction (Table 5) indicates different temporal patterns between species, with *R. nathaliae* reaching higher WUEi during late recovery (Supplementary Table S6A).

The A/Ci ratio increased progressively during recovery and was best described by a linear model (*R*^2^ = 0.76; Supplementary Table S4; Fig. 6c). It rose from approximately 0.002 at 6 h to 0.0105 in *R. nathaliae* and 0.013 in *R. serbica* at 168 h. Time had a highly significant effect, whereas species and the interaction were not significant (Table 5), indicating similar overall increase in A/Ci during rehydration (Supplementary Table S6B).

Overall, both *Ramonda* species exhibited strong and coordinated recovery of gas exchange after freezing stress. *Ramonda serbica* generally showed higher late-stage *A*, *g*_sw_ and *E* values, whereas *R. nathaliae* reached higher late-stage WUEi. These species-specific temporal patterns were consistent with the significant time × species interactions detected for *A*, Ci, *g*_sw_, *E* and WUEi, whereas *A*/Ci showed a similar temporal response in both species (Table 5).

## Discussion

Resurrection plants are classical models for desiccation tolerance, yet many previous studies rely on isolated traits or end-point measurements. By integrating chlorophyll fluorescence imaging, multispectral sensing and gas-exchange analysis, this study provides a time-resolved view of how *R. serbica* and *R. nathaliae* coordinate water relations, photosynthesis and photoprotection during cooling, freezing and recovery. Regression-based modelling additionally revealed turning points and recovery rates, offering a mechanistic understanding of freezing-induced desiccation and subsequent rehydration. Such an approach is particularly valuable for homoiochlorophyllous resurrection plants, which must maintain photochemical integrity despite extreme water loss. The physiological responses observed during the freezing phase should be interpreted as resulting from the combined effects of low temperature and dehydration rather than from temperature alone. As leaf water content declined markedly at subzero temperatures, part of the reduction in photosynthetic activity and associated optical traits likely reflected freeze-induced cellular dehydration in addition to direct thermal inhibition. This combined stress scenario is ecologically relevant for resurrection plants naturally exposed to winter freezing events (Gechev et al. 2013, Georgieva et al. 2020).

Declining RWC during cold and freezing served as the initial signal activating protective responses (Fig. 2; Table 2). Similar patterns have been described in *Ramonda* species (Gashi et al. 2013, Mihailova et al. 2023), reflecting osmotic adjustment and accumulation of compatible solutes such as sucrose, raffinose and proline. These metabolites stabilize membranes and proteins and support formation of the ‘glassy state’ typical of desiccation-tolerant tissues (Kuroki et al. 2019). The concurrent accumulation of dehydrins and ELIPs likely contributed to the stabilization of membranes as water content decreased, enabling plants to undergo controlled, freezing-induced desiccation rather than mechanical freezing injury (Georgieva et al. 2007, Fernández-Marín et al. 2018). During recovery, both species exhibited an initial slow rehydration phase, likely serving to avoid rapid osmotic influx and oxidative bursts associated with fast water uptake (Hura et al. 2018). Once this controlled phase passed, water uptake accelerated, indicating restoration of aquaporin activity and turgor (Gechev et al. 2013). No significant difference in RWC recovery was detected between the two species, suggesting that their overall capacity to restore leaf water status following freezing-induced desiccation was comparable under the experimental conditions (Fig. 2; Table 2). Thus, despite differences in their natural habitats, our results provide no evidence for species-specific differences in RWC recovery. CFTs revealed coordinated PSII adjustments under low-temperature stress. The decline in maximum PSII efficiency in the dark-adapted state (*F_v_*/*F_m_*) and effective PSII quantum yield in the light-adapted state (*F_q_*′/*F_m_*′) during freezing reflected reversible downregulation of PSII rather than permanent photodamage (Fig. 3; Table 3), consistent with responses observed in other resurrection plants during dehydration (Georgieva et al. 2007, Gechev et al. 2013). Such photoinhibition-like downregulation prevents excess excitation energy from over-reducing electron transport when carbon fixation is limited (Challabathula et al. 2018). Upon rehydration, both species rapidly restored PSII function, with broadly similar recovery patterns in the two species, as indicated by the absence of significant species and time × species effects for *F_v_*/*F_m_* and *F_q_*′/*F_m_*′ (Table 3). Although the fitted recovery curves showed some differences in their kinetic parameters, both species converged towards similar final values. Similar recovery patterns have been observed under natural low-temperature conditions (Gashi et al. 2025). Structural and biochemical adjustments that accompany rehydration including mesophyll remodelling, thylakoid reorganization and chloroplast expansion (Moore et al. 2007, Charuvi et al. 2019) likely contributed to the restoration of PSII efficiency in both species. It should be noted that our chlorophyll fluorescence parameters (including *F_v_*/*F_m_* and *F_q_*′/*F_m_*′) are whole-rosette averages derived from chlorophyll fluorescence imaging, inherently integrating spatial heterogeneity between young and old leaf sectors. Lower values at the rosette periphery, where aging and partially desiccated tissue accumulate, depress the canopy-level mean, whereas young central leaves showed *F_v_*/*F_m_* > 0.75. This likely explains why *F_v_*/*F_m_* values at the beginning of measurement (Phase I) as well as after the recovery (Phase VI) are lower than the ≥0.80 values reported for fully recovered *Ramonda* plants assessed by point measurements on selected healthy leaves (Mihailova et al. 2023, Kastrati et al. 2025).

Despite the concurrent reduction in PSII photochemical efficiency, a progressive decline in NPQ during cooling and freezing in both *Ramonda* species was found (Fig. 3e). This response contrasts with the enhancement of regulated thermal energy dissipation reported during seasonal cold acclimation of naturally growing *R. serbica* and *R. nathaliae* (Kastrati et al. 2025). The contrasting pattern may reflect differences between gradual seasonal acclimation under natural conditions and the sequential temperature exposure applied in the present controlled experiment. In our plants, NPQ declined together with photochemistry, consistent with broad downregulation of PSII function rather than a compensatory increase in rapidly inducible nonphotochemical energy dissipation. Therefore, the reduction in NPQ should not be interpreted as evidence of a lower requirement for photoprotection. At subzero temperatures, the decrease in NPQ coincided with pronounced freezing-induced water loss. A comparable nonlinear response was reported in *R. serbica*, where NPQ increased during moderate dehydration but declined after further water loss, followed by a transient increase during early rehydration (Augusti et al. 2001). Likewise, in *H. rhodopensis*, regulated NPQ is enhanced during moderate dehydration but becomes constrained under severe water loss, when PSII photochemistry is strongly inhibited and nonregulated energy dissipation becomes proportionally more important (Georgieva and Mihailova 2024). These observations suggest that the capacity for dynamically regulated energy dissipation may become constrained as dehydration intensifies, despite the continued need for photoprotection.

Upon rehydration, NPQ increased rapidly in both species while gas-exchange traits were still recovering, consistent with a transient photoprotective role during the early restoration of photosynthetic function (Augusti et al. 2001, Georgieva et al. 2020). During later recovery, *R. serbica* maintained higher NPQ values than *R. nathaliae* (Fig. 3f), consistent with the significant species and species × time effects observed for this trait; however, the underlying molecular mechanisms cannot be resolved from the present measurements.

Multispectral responses showed that pigment content and leaf structure responded in tightly coordinated but temporally decoupled ways. NDVI and specific green reflectance declined as temperatures approached freezing, indicating reduced apparent greenness and dehydration-induced changes in leaf optical properties (Fig. 4; Table 4), potentially linked to pigment adjustments. Logistic and quadratic trajectories suggest gradual, regulated shifts consistent with controlled desiccation rather than freeze shock. Early spectral shifts likely represent pre-emptive photoprotection via increased carotenoid and anthocyanin engagement (Fernández-Marín et al. 2020, Kastrati et al. 2025). During recovery, NDVI increased rapidly in both species, reflecting the fast restoration of pigment function. In contrast, specific green reflectance recovered more slowly, indicating delayed restoration of mesophyll structure, tissue turgor and water-related optical properties. Taken together, these results suggest that photosynthetic function can resume before complete restoration of leaf structural and optical properties associated with rehydration. While *F_v_*/*F_m_*, NDVI and net photosynthesis recovered relatively rapidly, specific green reflectance remained partially depressed, implying that mesophyll re-expansion, internal air-space organization and related scattering properties recover more gradually, as also reported for other resurrection plants during rehydration (Rascio and Rocca 2005, Gechev et al. 2013, Challabathula et al. 2016).

Cold exposure markedly reduced CO_2_ assimilation, stomatal conductance and transpiration, reflecting strong stomatal limitation and probable inhibition of RuBisCO (Fig. 5; Table 5). Similar responses have been described for *R. myconi* and *H. rhodopensis* under low temperature and dehydration (Georgieva et al. 2010, Fernández-Marín et al. 2020). *Ramonda serbica* maintained higher CO_2_ assimilation, stomatal conductance, transpiration and A/Ci values, indicating greater photosynthetic capacity per unit intercellular CO_2_. The increase in intercellular CO_2_ concentration with decreasing temperature, less pronounced in *R. serbica*, suggests more effective CO_2_ drawdown in this species. During recovery, both species showed a steady linear increase in CO_2_ assimilation (Fig. 6), consistent with rapid reactivation of photosynthesis observed under natural conditions (Gashi et al. 2025). The early decline in intercellular CO_2_ concentration, followed by stabilization, indicates re-engagement of RuBisCO carboxylation, with *R. serbica* exhibiting stronger initial CO_2_ utilization, aligning with its steeper A/Ci recovery. These patterns mirror field results showing dynamic coordination between stomatal opening and carbon fixation in *Ramonda* species (Kastrati et al. 2025). Stomatal conductance and transpiration exhibited nonlinear recovery, reflecting controlled reopening to limit water loss during early rehydration. *Ramonda serbica* tended to maintain higher stomatal conductance and transpiration later in recovery and showed more pronounced temporal changes, consistent with the significant time × species interactions for both traits (Table 5). These differences indicate species-dependent stomatal dynamics during recovery, although the underlying regulatory mechanisms cannot be resolved from the present measurements. Intrinsic water-use efficiency also showed species-dependent temporal dynamics during recovery, as indicated by the significant time × species interaction (Table 5). *Ramonda nathaliae* achieved higher late-stage intrinsic water-use efficiency, whereas *R. serbica* maintained comparatively lower values at the end of the recovery period. These differences suggest contrasting temporal regulation of carbon gain relative to stomatal water loss during recovery, although whether they represent distinct ecological water-use strategies requires further investigation. A limitation of this study is that the cold and freezing treatments were conducted under a constant 16/8 h light/dark photoperiod. This regime does not reflect the progressively shorter day lengths occurring at the Kosovo collection sites during autumn and winter. Since photoperiod may interact with temperature in the regulation of cold acclimation and freezing tolerance, the observed responses should be interpreted specifically as responses to sequential cold and freezing exposure under controlled growth-chamber conditions. They should therefore not be considered a complete representation of natural autumn–winter acclimation dynamics or overwintering performance. In addition, post-stress recovery was initiated by rehydration and transfer to favourable temperature conditions (20/15 °C). This approach enabled a standardized comparison of recovery capacity among treatments and species; however, it did not reproduce the gradual seasonal warming, variable soil–water conditions, or recurrent freeze–thaw events that plants may experience under natural conditions. Consequently, the recovery rates observed in this experiment should be interpreted as indicators of recovery potential rather than as direct estimates of recovery rates or survival performance in the field.

## Conclusions

This study shows that *R. serbica* and *R. nathaliae* share a largely reversible physiological response to sequential cooling, freezing-induced dehydration and subsequent rehydration. Both species retained a substantial capacity to restore PSII performance and progressively recover gas exchange, while species-dependent differences were most evident in NPQ and in the temporal trajectories of several gas-exchange traits during recovery. These differences indicate variation in the timing and magnitude of particular recovery processes rather than fundamentally different freezing-tolerance strategies. Importantly, because decreasing temperature, freezing and tissue dehydration occurred simultaneously, the observed physiological changes should be interpreted as an integrated response to interacting low-temperature and dehydration stresses rather than as responses specific to freezing alone.

Several limitations define the scope of these conclusions. Controlled growth-chamber conditions simplified the environmental complexity experienced by plants in their natural habitats, including seasonal changes in photoperiod, fluctuating water availability and repeated freeze–thaw events. Leaf rolling prevented gas-exchange measurements during freezing and the earliest stages of recovery, limiting direct comparison between photochemical and carbon-assimilation responses during this critical transition. Furthermore, fluorescence and multispectral imaging provide noninvasive functional proxies but do not directly identify the biochemical or molecular processes underlying stress tolerance and recovery. Future work should therefore combine experimental separation of temperature and dehydration effects with repeated freeze–thaw treatments, more seasonally realistic environmental regimes and comparisons among multiple populations.

An important methodological extension of the present whole-rosette phenotyping approach would be to resolve spatial variation within individual rosettes. Our observations suggest that younger, inner leaves may retain physiological activity longer during freezing and recover more rapidly after rehydration than older, outer leaves. Quantifying these within-plant differences using spatially resolved fluorescence and multispectral phenotyping, coupled with targeted measurements of water relations, cellular injury and biochemical and molecular responses in leaves of different developmental positions, could reveal whether recovery is spatially coordinated within the rosette. Such an approach would move beyond whole-plant averages and help identify the tissues and processes that are most important for maintaining viability and re-establishing photosynthetic function after freezing-induced dehydration.

## Supplementary Material

plag046_Supplementary_Data

## Data Availability

The data underlying the results presented in this study are openly available in Zenodo at https://doi.org/10.5281/zenodo.22749118 The repository contains the observation-level data used in the analyses together with a README file describing the dataset structure, variables, and information required to reproduce the statistical analyses. Statistical analyses were conducted using JMP software; no custom analysis code was used.
