# Supplementary material for: Integrated phenotyping reveals coordinated physiological responses to cold, freezing, and recovery in Ramonda resurrection plants
Source: AoB Plants. 2026 Sep 29;18(5):plag046. doi: 10.1093/aobpla/plag046 (PMC13659235; doi:10.1093/aobpla/plag046)
Supplement: Supplementary file 1 [file plag046_supplementary_data.pdf]

Supplementary Table 1. Variable clustering of chlorophyll fluorescence traits (CFT)

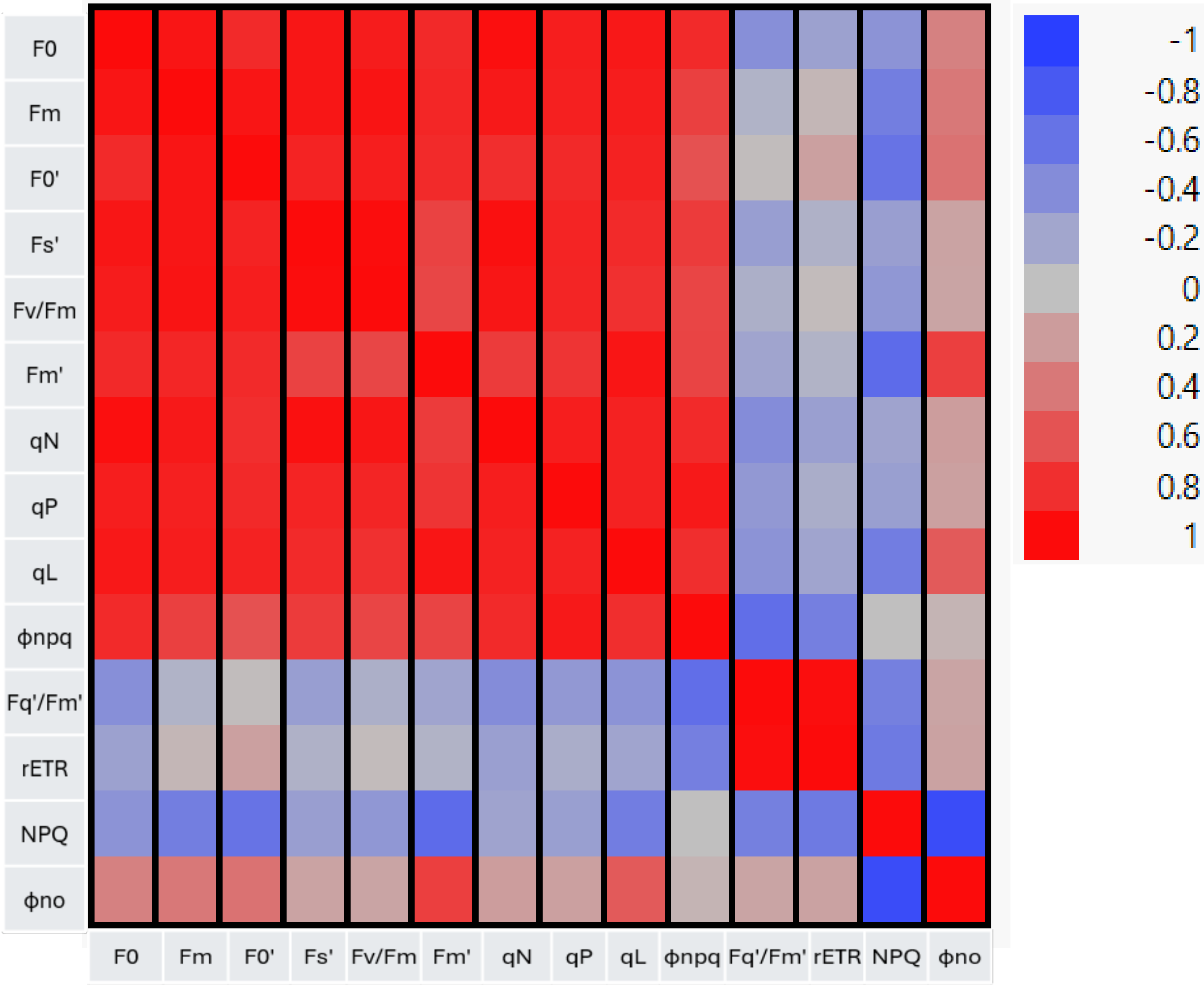

Cluster summary

Cluster	Number of	Most Representative V	Cluster Proportion of Variation I	Total Proportion of Variation Explained
1	10	F0	0.867	0.578
2	2	Fq'/Fm'	0.916	0.183
3	2	NPQ	0.948	0.126

Cluster members

Cluster	Members	RSquare with Own Clu	RSquare with Next Closest	1-RSquare Ratio
1	F0	0.954	0.129	0.052
1	Fm	0.946	0.218	0.069
1	F0'	0.93	0.046	0.073
1	Fs'	0.905	0.047	0.099
1	Fv/Fm	0.89	0.05	0.116
1	Fm'	0.877	0.06	0.131
1	qN	0.897	0.309	0.149
1	qP	0.835	0.278	0.229
1	qL	0.7	0.238	0.393
1	ϕnpq	0.739	0.5	0.521
2	Fq'/Fm'	0.98	0.134	0.024
2	rETR	0.933	0.112	0.075
3	NPQ	0.948	0.118	0.059
3	ϕno	0.948	0.307	0.075

Supplementary Table 2. Variable clustering of multispectral traits (MST)

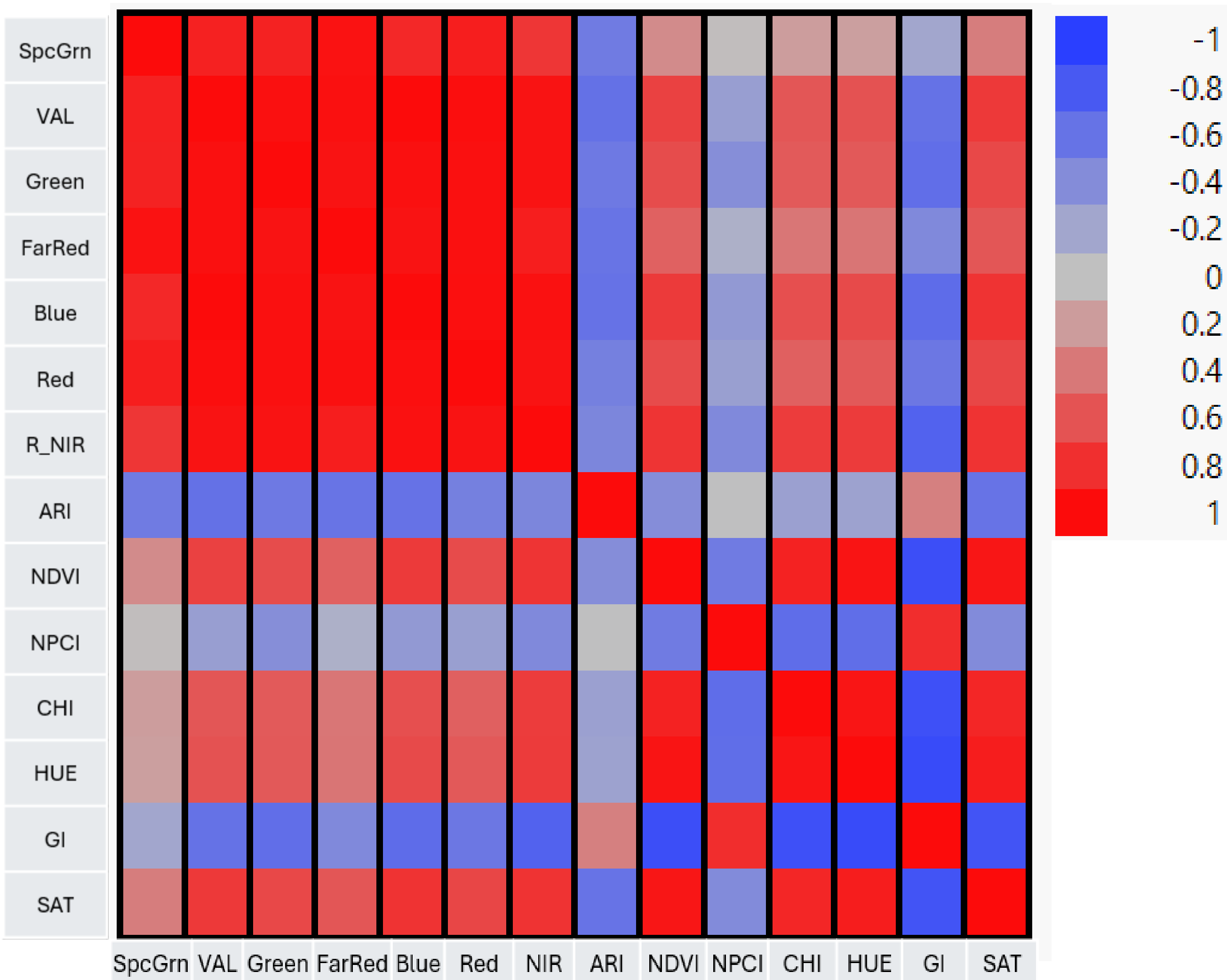

Cluster summary

Cluster	Number of	Most Representative V	Cluster Proportion of Variation I	Total Proportion of Variation Explained
1	8	R_SpcGrn	0.872	0.498
2	6	NDVI	0.84	0.36

Cluster members

Cluster	Members	RSquare with Own Clu	RSquare with Next Closest	1-RSquare Ratio
1	R_SpcGrn	0.99	0.421	0.018
1	VAL	0.971	0.215	0.037
1	R_Green	0.976	0.481	0.046
1	R_FarRed	0.97	0.358	0.047
1	R_Blue	0.963	0.401	0.061
1	R_Red	0.828	0.053	0.182
1	R_NIR	0.892	0.589	0.264
1	ARI	0.388	0.117	0.693
2	NDVI	0.956	0.293	0.063
2	NPCI	0.929	0.319	0.105
2	CHI	0.9	0.281	0.139
2	HUE	0.906	0.407	0.159
2	GI	0.835	0.483	0.32
2	SAT	0.516	0.06	0.515

Supplementary Table 3. Model performance comparison for different regression types applied during cold and freezing period for response of relative water content (RWC), chlorophyll fluorescence traits (Maximum Efficiency of PSII (Fv/Fm; Effective Quantum Yield of PSII (Fq'/Fm'); Non-Photochemical Quenching (NPQ)) and multispectral traits (Specific Green Reflection (RSpcGrn) and Normalised Difference Vegetation Index (NDVI).

Trait	Model	AICc	AICc Weight	BIC	SSE	MSE	RMSE	R-Square
RWC	**Logistic 3P**	216.74	0.999	221.46	1276.24	53.18	7.29	0.957
	Quadratic	232.36	0.000	237.07	2147.79	89.49	9.46	0.928
	Exponential 3P	234.88	0.000	239.60	2336.38	97.35	9.87	0.922
	Linear	242.88	0.000	247.38	3799.32	146.13	12.09	0.873
	Exponential 2P	260.87	0.000	265.38	6921.79	266.22	16.32	0.769
Fv/Fm	**Quadratic**	-363.58	0.588	-345.06	0.300	0.003	0.051	0.894
	Exponential 3P	-361.65	0.224	-343.14	0.304	0.003	0.052	0.892
	Exponential 2P	-355.37	0.010	-341.96	0.333	0.003	0.054	0.882
	Logistic 3P	-350.56	0.001	-332.05	0.334	0.003	0.054	0.882
	Linear	-320.21	0.000	-306.79	0.446	0.004	0.062	0.842
Fq'/Fm'	**Linear**	-459.45	0.102	-446.03	0.140	0.001	0.035	0.659
	Exponential 2P	-456.78	0.027	-443.37	0.143	0.001	0.035	0.652
	Logistic 3P	-456.37	0.022	-437.86	0.138	0.001	0.035	0.663
	Quadratic	-455.03	0.011	-436.52	0.140	0.001	0.035	0.660
	Exponential 3P	-455.02	0.011	-436.51	0.140	0.001	0.035	0.660
NPQ	**Logistic 3P**	-144.83	0.991	-126.31	1.855	0.016	0.128	0.936
	Quadratic	-135.39	0.009	-116.87	2.007	0.018	0.133	0.931
	Exponential 3P	-121.70	0.000	-103.18	2.249	0.020	0.140	0.923
	Exponential 2P	-117.55	0.000	-104.14	2.416	0.021	0.144	0.917
	Linear	-81.79	0.000	-68.38	3.255	0.028	0.168	0.888
RSpcGrn	**Logistic 3P**	1612.71	0.006	1631.22	4256031.90	37333.61	193.22	0.821
	Linear	1615.63	0.001	1629.05	4526754.90	39023.75	197.54	0.810
	Quadratic	1618.42	0.000	1636.93	4463411.30	39152.73	197.87	0.813
	Exponential 3P	1618.76	0.000	1637.28	4476324.90	39266.01	198.16	0.812
	Exponential 2P	1630.23	0.000	1643.64	5112201.10	44070.70	209.93	0.785
NDVI	**Quadratic**	-407.78	0.480	-389.27	0.207	0.002	0.043	0.829
	Logistic 3P	-407.38	0.392	-388.86	0.208	0.002	0.043	0.828
	Exponential 3P	-405.13	0.128	-386.62	0.212	0.002	0.043	0.825
	Linear	-383.06	0.000	-369.65	0.264	0.002	0.048	0.782
	Exponential 2P	-372.43	0.000	-359.01	0.289	0.002	0.050	0.761

Supplementary Table 4. Model performance comparison for different regression types applied during recovery period for response of relative water content (RWC), chlorophyll fluorescence traits (Maximum Efficiency of PSII (Fv/Fm; Effective Quantum Yield of PSII (Fq'/Fm'); Non-Photochemical Quenching (NPQ)), multispectral traits (Specific Green Reflection (RSpcGrn) and Normalised Difference Vegetation Index (NDVI)) and gas exchange traits (net CO_2 assimilation rate (A); stomatal conductance (gsw); transpiration rate (E); intercellular CO_2 concentration (Ci); intrinsic water use efficiency (WUEi); A/Ci ratio.

Trait	Model	AICc	AICc Weight	BIC	SSE	MSE	RMSE	R-Square
**RWC**	**Logistic 3P**	**320.41398**	**1**	**331.90208**	**880.37799**	**18.341208**	**4.2826637**	**0.9789186**
	Exponential 3P	356.74	0.000	368.23	1725.11	35.94	5.99	0.959
	Quadratic	378.18	0.000	389.67	2566.02	53.46	7.31	0.939
	Linear	473.25	0.000	481.94	16426.52	328.53	18.13	0.607
	Exponential 2P	486.96	0.000	495.65	21173.66	423.47	20.58	0.493
**Fv/Fm**	**Logistic 3P**	-865.24	0.506	-842.15	0.215	0.001	0.032	0.931
	Quadratic	-862.08	0.104	-838.99	0.218	0.001	0.032	0.930
	Exponential 3P	-848.19	0.000	-825.10	0.233	0.001	0.033	0.926
	Linear	-665.89	0.000	-649.29	0.553	0.003	0.051	0.823
	Exponential 2P	-599.31	0.000	-582.72	0.752	0.004	0.060	0.760
**Fq'/Fm'**	**Logistic 3P**	-1071.49	0.544	-1048.40	0.083	0.000	0.020	0.886
	Quadratic	-1071.11	0.451	-1048.03	0.083	0.000	0.020	0.885
	Exponential 3P	-1062.09	0.005	-1039.00	0.087	0.000	0.020	0.880
	Linear	-898.99	0.000	-882.40	0.188	0.001	0.030	0.741
	Exponential 2P	-851.14	0.000	-834.55	0.234	0.001	0.033	0.676
**NPQ**	**Logistic 3P**	-297.49	0.354	-274.40	2.983	0.014	0.119	0.928
	Quadratic	-273.72	0.000	-250.63	3.330	0.016	0.126	0.919
	Exponential 3P	-224.08	0.000	-200.99	4.190	0.020	0.141	0.898
	Linear	-24.27	0.000	-7.68	10.777	0.051	0.225	0.738
	Exponential 2P	58.72	0.000	75.31	15.826	0.075	0.273	0.615
**RSpcGrn**	**Logistic 3P**	2635.77	0.874	2658.86	2356637.80	11222.09	105.93	0.82
	Exponential 3P	2639.65	0.126	2662.74	2399348.90	11425.47	106.89	0.82
	Quadratic	2665.74	0.000	2688.82	2707297.90	12891.90	113.54	0.80
	Linear	2827.49	0.000	2844.08	5838591.80	27540.53	165.95	0.56
	Exponential 2P	2852.30	0.000	2868.89	6549346.00	30893.14	175.76	0.51
**NDVI**	**Logistic 3P**	-901.87	0.839	-877.98	0.309	0.001	0.036	0.814
	Exponential 3P	-898.56	0.161	-874.68	0.313	0.001	0.037	0.812
	Quadratic	-832.99	0.000	-809.11	0.411	0.002	0.042	0.753
	Linear	-667.13	0.000	-649.98	0.836	0.004	0.060	0.498
	Exponential 2P	-654.48	0.000	-637.33	0.881	0.004	0.061	0.470
	**Linear**	97.90	0.765	106.83	15.421	0.297	0.545	0.737

**A**	Exponential 3P	102.45	0.079	114.29	15.260	0.305	0.552	0.740
	Quadratic	102.46	0.078	114.30	15.263	0.305	0.553	0.740
	Logistic 3P	103.21	0.054	115.05	15.469	0.309	0.556	0.736
	Exponential 2P	104.77	0.025	113.70	17.433	0.335	0.579	0.703
**gsw**	**Quadratic**	-86.60	0.801	-74.76	0.522	0.010	0.102	0.770
	Exponential 3P	-83.81	0.198	-71.97	0.548	0.011	0.105	0.758
	Exponential 2P	-71.54	0.000	-62.61	0.748	0.014	0.120	0.670
	Logistic 3P	-66.32	0.000	-54.47	0.749	0.015	0.122	0.670
	Linear	-45.47	0.000	-36.55	1.192	0.023	0.151	0.475
**E**	**Quadratic**	131.41	0.816	143.26	25.596	0.512	0.715	0.774
	Exponential 3P	134.39	0.184	146.24	26.995	0.540	0.735	0.762
	Exponential 2P	146.67	0.000	155.60	36.842	0.709	0.842	0.675
	Logistic 3P	151.78	0.000	163.62	36.824	0.736	0.858	0.675
	Linear	173.28	0.000	182.20	59.247	1.139	1.067	0.477
**Ci**	**Quadratic**	403.44	0.866	415.28	3294.56	65.891	8.117	0.847
	Exponential 3P	410.44	0.026	422.28	3733.20	74.664	8.641	0.827
	Exponential 2P	449.25	0.000	458.18	8183.24	157.370	12.545	0.621
	Linear	451.87	0.000	460.79	8574.09	164.886	12.841	0.603
	Logistic 3P	454.39	0.000	466.23	8183.31	163.666	12.793	0.621
**WUEi**	**Logistic 3P**	272.43	0.409	284.28	317.56	6.351	2.520	0.844
	Exponential 3P	273.71	0.216	285.55	324.88	6.498	2.549	0.840
	Quadratic	273.75	0.211	285.60	325.13	6.503	2.550	0.840
	Linear	274.27	0.163	283.19	359.64	6.916	2.630	0.823
	Exponential 2P	295.50	0.000	304.42	525.41	10.104	3.179	0.742
**A/Ci**	**Linear**	-550.24	0.741	-541.31	0.000	0.000	0.002	0.761
	Exponential 3P	-545.98	0.088	-534.13	0.000	0.000	0.002	0.765
	Quadratic	-545.98	0.088	-534.13	0.000	0.000	0.002	0.765
	Logistic 3P	-545.25	0.061	-533.40	0.000	0.000	0.002	0.762
	Exponential 2P	-540.43	0.006	-531.50	0.000	0.000	0.002	0.716

Supplementary Table 5 A Estimated marginal means (EMM ± SE) and compact letter display (CLD) for relative water content (RWC), chlorophyll fluorescence parameters (Fv/Fm, Fq'/Fm', NPQ), multispectral parameters (R_SpcGrn and NDVI) of *Ramonda nathaliae* and *R. serbica* measured during the cooling and freezing phases at 20, 10, 0, −5, and −10 °C.

Means sharing the same letter within each species are not significantly different according to Tukey-adjusted post hoc comparisons ($P < 0.05$).

Supplementary table 5 B Sliced species comparisons at each temperature level for relative water content (RWC), chlorophyll fluorescence parameters (Fv/Fm, Fq'/Fm', NPQ), multispectral parameters (R_SpcGrn and NDVI) during the cooling treatment. P-values represent pairwise contrasts between species (*R. serbica* − *R. nathaliae*) obtained from linear mixed-effects models. Significant differences ($P < 0.05$) are shown in bold.

A)

	**RWC**				**Fv/Fm**				**Fq'/Fm'**			
	R nathaliae		*R. serbica*		*R nathaliae*		*R. serbica*		*R nathaliae*		*R. serbica*	
**Temperature (°C)**	**EMM ± SE**	**CLD**	**EMM ± SE**	**CLD**	**EMM ± SE**	**CLD**	**EMM ± SE**	**CLD**	**EMM ± SE**	**CLD**	**EMM ± SE**	**CLD**
20	88.34 ± 2.57	a	91.57 ± 2.57	a	0.64 ± 0.014	a	0.67 ± 0.014	a	0.312 ± 0.020	a	0.392 ± 0.008	a
10	81.00 ± 2.57	a	75.41 ± 2.57	b	0.43 ± 0.014	b	0.47 ± 0.014	b	0.239 ± 0.010	b	0.283 ± 0.004	b
0	56.64 ± 2.57	b	60.48 ± 2.57	c	0.29 ± 0.014	c	0.32 ± 0.014	c	0.161 ± 0.011	b	0.195 ± 0.006	b
-5	16.18 ± 2.57	c	20.15 ± 2.57	d	0.27 ± 0.014	c	0.30 ± 0.014	c	0.125 ± 0.007	b	0.158 ± 0.010	b
-10	12.78 ± 2.57	c	11.94 ± 2.57	d	0.26 ± 0.014	c	0.28 ± 0.014	c	0.09 ± 0.010	b	0.110 ± 0.004	b

	**NPQ**				**R_SpcGrn**				**NDVI**			
	R nathaliae		*R. serbica*		*R nathaliae*		*R. serbica*		*R nathaliae*		*R. serbica*	
**Temperature (°C)**	**EMM ± SE**	**CLD**	**EMM ± SE**	**CLD**	**EMM ± SE**	**CLD**	**EMM ± SE**	**CLD**	**EMM ± SE**	**CLD**	**EMM ± SE**	**CLD**
20	1.238 ± 0.068	a	1.687 ± 0.065	a	1741 ± 109	a	1788 ± 34	a	0.783 ± 0.009	a	0.786 ± 0.004	a
10	0.775 ± 0.033	b	1.121 ± 0.041	b	1506 ± 73	b	1570 ± 23	b	0.740 ± 0.012	a	0.756 ± 0.004	a
0	0.360 ± 0.008	c	0.371 ± 0.009	c	1120 ± 64	c	1284 ± 46	c	0.660 ± 0.017	b	0.677 ± 0.011	b
-5	0.281 ± 0.005	c	0.260 ± 0.010	c	771 ± 18	c	835 ± 29	d	0.582 ± 0.014	c	0.604 ± 0.016	c
-10	0.245 ± 0.003	c	0.211 ± 0.011	c	446 ± 23	c	480 ± 24	d	0.520 ± 0.012	c	0.536 ± 0.004	c

B)

Sliced species comparisons at each temperature

			**p value**			
Temperature (°C)	**RWC**	**Fv/Fm**	**Fq'/Fm'**	**NPQ**	**R_SpcGrn**	**NDVI**
20	0.386	0.1403	0.08	**<0.001**	0.689	0.752
10	0.139	0.0859	0.424	**<0.001**	0.645	0.195
0	0.303	0.1403	0.371	**0.02**	0.423	0.385
-5	0.288	0.2184	0.407	**<0.001**	0.061	0.124
-10	0.819	0.2184	0.424	**<0.001**	0.645	0.195

Supplementary table 6 A Estimated marginal means (EMM ± SE) and compact letter display (CLD) for relative water content (RWC), chlorophyll fluorescence parameters (Fv/Fm, Fq'/Fm', NPQ), multispectral parameters (R_SpcGrn and NDVI), and gas exchange parameters (A, E, gsw, Ci, A/Ci, WUEi) of Ramonda nathaliae and R. serbica measured during the recovery phase.

Means sharing the same letter within each species are not significantly different according to Tukey-adjusted post hoc comparisons (P < 0.05).

Supplementary table 6 B Sliced species comparisons at each timepoint during the recovery phase for relative water content (RWC), chlorophyll fluorescence parameters (Fv/Fm, Fq'/Fm', NPQ), multispectral parameters (RSpcGrn and NDVI) and gas exchange parameters (A, E, gsw, Ci, A/Ci, WUEi) during the recovery phase. P-values represent pairwise contrasts between species (R. serbica − R. nathaliae) obtained from linear mixed-effects models.
Significant differences (P < 0.05) are shown in bold.

	**RWC**				**Fv/Fm**				**Fq'/Fm'**			
	R nathaliae		*R. serbica*		*R nathaliae*		*R. serbica*		*R nathaliae*		*R. serbica*	
**Time (h)**	**EMM ± SE**	**CLD**	**EMM ± SE**	**CLD**	**EMM ± SE**	**CLD**	**EMM ± SE**	**CLD**	**EMM ± SE**	**CLD**	**EMM ± SE**	**CLD**
1	15.6 ± 4.64	d	13.8 ± 2.24	d	0.215 ± 0.016	d	0.201 ± 0.019	d	0.051 ± 0.011	d	0.058 ± 0.010	d
3	21.9 ± 2.55	d	25.5 ± 6.81	cd	0.225 ± 0.021	cd	0.221 ± 0.018	cd	0.078 ± 0.013	cd	0.076 ± 0.011	cd
6	30.8 ± 2.85	cd	22.3 ± 1.25	d	0.262 ± 0.017	c	0.251 ± 0.015	c	0.092 ± 0.012	c	0.091 ± 0.010	c
9	32.5 ± 2.31	c	35.1 ± 4.47	c	0.281 ± 0.014	bc	0.278 ± 0.013	bc	0.119 ± 0.010	bc	0.118 ± 0.009	bc
12	49.2 ± 6.48	b	38.3 ± 2.68	c	0.311 ± 0.013	b	0.314 ± 0.012	b	0.121 ± 0.010	b	0.129 ± 0.009	b
24	65.8 ± 3.34	b	58.9 ± 2.34	b	0.353 ± 0.011	ab	0.357 ± 0.011	ab	0.152 ± 0.009	ab	0.161 ± 0.008	ab
48	80.8 ± 2.64	a	85.0 ± 2.16	a	0.405 ± 0.010	a	0.464 ± 0.009	a	0.266 ± 0.008	a	0.243 ± 0.007	a
72	82.6 ± 1.37	a	86.9 ± 2.20	a	0.562 ± 0.009	a	0.621 ± 0.008	a	0.341 ± 0.007	a	0.301 ± 0.007	a
168	86.5 ± 2.85	a	87.7 ± 2.06	a	0.689 ± 0.008	a	0.694 ± 0.007	a	0.381 ± 0.007	a	0.367 ± 0.006	a

	**NPQ**				**R_SpcGrn**				**NDVI**			
	R nathaliae		*R. serbica*		*R nathaliae*		*R. serbica*		*R nathaliae*		*R. serbica*	
**Time (h)**	**EMM ± SE**	**CLD**	**EMM±SE**	**CLD**	**EMM±SE**	**CLD**	**EMM±SE**	**CLD**	**EMM ± SE**	**CLD**	**EMM ± SE**	**CLD**
1	0.311 ± 0.021	d	0.287 ± 0.023	d	515 ± 28	d	548 ± 26	d	0.51 ± 0.012	d	0.50 ± 0.011	d
3	0.328 ± 0.028	d	0.302 ± 0.027	d	602 ± 31	d	638 ± 29	d	0.52 ± 0.013	d	0.53 ± 0.012	cd
6	0.366 ± 0.031	c	0.301 ± 0.031	d	745 ± 34	cd	779 ± 32	cd	0.53 ± 0.014	cd	0.54 ± 0.013	c
9	0.434 ± 0.035	bc	0.412 ± 0.034	cd	765± 39	cd	814± 39	c	0.54 ± 0.012	c	0.55 ± 0.011	c
12	0.461 ± 0.041	b	0.431 ± 0.039	bc	798 ± 31	c	831± 35	c	0.57 ± 0.011	c	0.58 ± 0.010	bc
24	0.615 ± 0.046	b	0.722 ± 0.042	b	986 ± 34	b	1012± 39	b	0.66 ± 0.012	b	0.65 ± 0.011	b
48	0.772 ± 0.051	ab	1.173 ± 0.046	ab	1128 ± 42	a	1185 ± 47	a	0.72 ± 0.009	a	0.71 ± 0.008	a
72	0.957 ± 0.048	a	1.514 ± 0.045	a	1216 ± 69	a	1255 ± 56	a	0.73 ± 0.008	a	0.72 ± 0.008	a
168	1.104 ± 0.047	a	1.628 ± 0.044	a	1268 ± 65	a	1301 ± 82	a	0.75 ± 0.008	a	0.73 ± 0.007	a

	**A (µmol CO_2 m ^{2} s ^{1})**				**Ci (µmol mol ^{1})**				**gsw (mol H_2O m ^{2} s ^{1})**			
	R nathaliae		*R. serbica*		*R nathaliae*		*R. serbica*		*R nathaliae*		*R. serbica*	
**Time (h)**	**EMM ± SE**	**CLD**	**EMM ± SE**	**CLD**	**EMM±SE**	**CLD**	**EMM±SE**	**CLD**	**EMM ± SE**	**CLD**	**EMM ± SE**	**CLD**
1	NA	NA	NA	NA	NA	NA	NA	NA	NA	NA	NA	NA
3	NA	NA	NA	NA	NA	NA	NA	NA	NA	NA	NA	NA
6	0.59 ± 0.22	d	0.68 ± 0.19	d	367.6 ± 1.11	a	365.0 ± 2.68	a	0.55 ± 0.02	ab	0.68 ± 0.08	a
9	0.87 ± 0.18	d	0.77 ± 0.15	d	364.4 ± 2.86	a	362.4 ± 2.19	a	0.56 ± 0.03	ab	0.74 ± 0.06	a
12	1.32 ± 0.06	cd	1.22 ± 0.03	cd	364.0 ± 0.89	a	363.2 ± 0.75	a	0.70 ± 0.02	a	0.62 ± 0.05	a
24	1.43 ± 0.19	cd	1.32 ± 0.12	cd	362.8 ± 3.24	a	347.9 ± 3.44	b	0.60 ± 0.06	ab	0.46 ± 0.05	b
48	1.65 ± 0.28	c	1.79 ± 0.32	c	340.5 ± 2.48	b	336.3 ± 6.30	bc	0.36 ± 0.04	bc	0.27 ± 0.03	c
72	1.84 ± 0.18	bc	2.32 ± 0.25	b	334.2 ± 3.85	b	314.4 ± 4.69	c	0.19 ± 0.01	cd	0.26 ± 0.04	c
168	3.51 ± 0.67	a	4.02 ± 0.20	a	335.6 ± 2.41	b	310.4 ± 4.36	c	0.17 ± 0.02	d	0.29 ± 0.05	c

	**E (mmol H_2O m ^{2} s ^{1})**				**WUEi (µmol CO_2 mmol ^{1} H_2O)**				**A/Ci**			
	R nathaliae		*R. serbica*		*R nathaliae*		*R. serbica*		*R nathaliae*		*R. serbica*	
**Time (h)**	**EMM ± SE**	**CLD**	**EMM ± SE**	**CLD**	**EMM ± SE**	**CLD**	**EMM ± SE**	**CLD**	**EMM ± SE**	**CLD**	**EMM ± SE**	**CLD**
1	NA	NA	NA	NA	NA	NA	NA	NA	NA	NA	NA	NA
3	NA	NA	NA	NA	NA	NA	NA	NA	NA	NA	NA	NA
6	3.75 ± 0.12	ab	4.76 ± 0.59	a	1.08 ± 0.43	d	0.97 ± 0.21	d	0.0016 ± 0.00061	d	0.0019 ± 0.00053	d
9	4.24 ± 0.26	a	5.15 ± 0.42	a	1.55 ± 0.27	d	1.08 ± 0.27	d	0.0024 ± 0.00051	cd	0.0021 ± 0.00039	d
12	4.89 ± 0.10	a	4.32 ± 0.35	a	1.89 ± 0.08	d	2.17 ± 0.12	d	0.0036 ± 0.00017	c	0.0036 ± 0.00010	c
24	4.17 ± 0.41	a	3.24 ± 0.34	b	2.38 ± 0.14	d	3.93 ± 0.65	c	0.0039 ± 0.00055	c	0.0038 ± 0.00034	c
48	2.55 ± 0.24	bc	1.86 ± 0.20	c	4.80 ± 1.15	c	6.79 ± 0.88	b	0.0049 ± 0.00083	bc	0.0053 ± 0.00095	b
72	1.33 ± 0.10	cd	1.83 ± 0.25	c	10.08 ± 1.33	b	9.36 ± 1.03	b	0.0056 ± 0.00060	b	0.0074 ± 0.00088	b
168	1.19 ± 0.13	d	2.00 ± 0.34	c	20.86 ± 3.18	a	14.72 ± 2.14	a	0.0105 ± 0.00203	a	0.0129 ± 0.00064	a

B)
Sliced species comparisons at each timepoint

	**p value**											
Time (h)	**RWC**	**Fv/Fm**	**Fq'/Fm'**	**NPQ**	**RSpcGrn**	**NDVI**	**A**	**Ci**	**gsw**	**E**	**WUEi**	**A/Ci**
1	0.736	0.41	0.298	0.188	0.551	0.467	NA	NA	NA	NA	NA	NA
3	0.656	0.39	0.521	0.266	0.623	0.352	NA	NA	NA	NA	NA	NA
6	0.053	0.09	0.214	0.414	0.441	0.281	0.782	0.44	0.118	0.12	0.864	0.78
9	0.635	0.28	0.438	0.294	0.529	0.307	0.673	0.61	0.094	0.09	0.21	0.68
12	0.192	0.33	0.344	0.142	0.618	0.356	0.991	0.53	0.281	0.28	0.061	0.96
24	0.166	0.22	0.418	0.072	0.271	0.411	0.677	0.75	0.061	0.06	0.074	0.85
48	0.284	0.51	0.612	**0.008**	0.194	0.533	0.754	0.07	0.058	0.06	0.208	0.73
72	0.179	0.58	0.709	**0.003**	0.312	0.621	0.161	**0.01**	0.109	0.11	0.676	0.11
168	0.744	0.69	0.801	**0.015**	0.487	0.744	0.51	**0.01**	0.124	0.12	0.172	0.32
